# Structure of general-population antibody titer distributions to influenza A virus

**DOI:** 10.1038/s41598-017-06177-0

**Published:** 2017-07-20

**Authors:** Nguyen Thi Duy Nhat, Stacy Todd, Erwin de Bruin, Tran Thi Nhu Thao, Nguyen Ha Thao Vy, Tran Minh Quan, Dao Nguyen Vinh, Janko van Beek, Pham Hong Anh, Ha Minh Lam, Nguyen Thanh Hung, Nguyen Thi Le Thanh, Huynh Le Anh Huy, Vo Thi Hong Ha, Stephen Baker, Guy E. Thwaites, Nguyen Thi Nam Lien, Tran Thi Kim Hong, Jeremy Farrar, Cameron P. Simmons, Nguyen Van Vinh Chau, Marion Koopmans, Maciej F. Boni

**Affiliations:** 1 Wellcome Trust Major Overseas Programme, Oxford University Clinical Research Unit, Ho Chi Minh City, Vietnam; 2 Centre for Tropical Medicine, Nuffield Department of Clinical, Medicine, University of Oxford, Oxford, United Kingdom; 3 Liverpool School of Tropical Medicine, Liverpool, United Kingdom; 4 Department of Viroscience, Erasmus Medical Centre, Rotterdam, Netherlands; 5 National Institute for Public Health and the Environment, Bilthoven, Netherlands; 6 Khanh Hoa Provincial Hospital, Nha Trang, Vietnam; 7 Hospital for Tropical Diseases, Ho Chi Minh City, Vietnam; 8 Hue Provincial Hospital, Thua Thien Hue province, Vietnam; 9 Dak Lak General Hospital, Buon Ma Thuot, Vietnam; 10 The Wellcome Trust, London, UK; 11 Microbiology and Immunology Department, University of Melbourne, Melbourne, Australia; 12 Center for Infectious Disease Dynamics, Department of Biology, Pennsylvania State University, University Park, Pennsylvania USA

## Abstract

Seroepidemiological studies aim to understand population-level exposure and immunity to infectious diseases. Their results are normally presented as binary outcomes describing the presence or absence of pathogen-specific antibody, despite the fact that many assays measure continuous quantities. A population’s natural distribution of antibody titers to an endemic infectious disease may include information on multiple serological states – naiveté, recent infection, non-recent infection, childhood infection – depending on the disease in question and the acquisition and waning patterns of immunity. In this study, we investigate 20,152 general-population serum samples from southern Vietnam collected between 2009 and 2013 from which we report antibody titers to the influenza virus HA1 protein using a continuous titer measurement from a protein microarray assay. We describe the distributions of antibody titers to subtypes 2009 H1N1 and H3N2. Using a model selection approach to fit mixture distributions, we show that 2009 H1N1 antibody titers fall into four titer subgroups and that H3N2 titers fall into three subgroups. For H1N1, our interpretation is that the two highest-titer subgroups correspond to recent and historical infection, which is consistent with 2009 pandemic attack rates. Similar interpretations are available for H3N2, but right-censoring of titers makes these interpretations difficult to validate.

## Introduction

The distribution of antibodies in a human population is a fossil imprint of the population’s past exposure to infectious disease. If individuals’ antibody concentrations can be measured accurately, they can be used to infer both the size and timing of past epidemics. The two key post-epidemic processes that need to be measured to make this inference possible are the rate of antibody acquisition and the rate of antibody waning. The rate of antibody acquisition post-infection is rapid (weeks) for most viral pathogens, but more difficult to measure for more complex pathogens that present the immune system with a diverse set of antigens. The rate of antibody waning, however, is rarely measured even for viral pathogens. To correctly translate a population’s antibody titer distribution to its epidemic history, accurate measures of both these rates are necessary. To validate that this reconstruction has been done correctly, a large cohort with long-term follow-up and precise antibody measurements would be required. Studies like these are difficult to run and difficult to find in the scientific literature – both in methodological development and field implementation. Further complicating the issue is that antibody measurements are rarely 100% specific, and that low-level cross-reactive antibodies often are ignored by setting a cut-off for positivity.

To begin investigating what an antibody distribution can tell us about a population’s epidemic history, we initiated a large-scale time-structured serological survey^1, 2^ and an observational clinical study that includes repeat patient follow-ups to measure rates of antibody waning^3^; the results of the serological survey are presented here. Influenza A virus was chosen as the pathogen of interest as (*i*) it is an important, globally-circulating human pathogen, (*ii*) influenza is well characterized antigenically, (*iii*) a precise and repeatable serological assay was available, and (*iv*) the human population receives almost no influenza vaccination in our study location of southern Vietnam. The first aim of this study was to move away from the binary approach to serology – which classifies individuals as seropositive or seronegative^4–8^ – and to describe the underlying structure of a general-population antibody-titer distribution by assuming that an individual can belong to any number of serological states.

The rationale for a detailed descriptive analysis of antibody titer distributions is that titer groups or titer ranges may be able to provide differentiating information on the type of infection, e.g. recent versus non-recent infections, or primary versus non-primary. The binary approach of classifying individuals as seropositive and seronegative is not as informative as it could be given the richness of some serological datasets, and it is already known to have two practical drawbacks. First, the cutoff value for seropositivity is typically calibrated from a group of patients with confirmed acute infection, by collecting convalescent serum samples a few weeks or a few months after symptoms onset. This means that the correct application of the cut-off value is the identification of recent symptomatic infections rather than any past infections. Thus, applying this threshold to a population-wide serological cross-section will likely result in an underestimate of the seroprevalence. Second, binary classification in serology results in incorrect or inconclusive classifications for samples with borderline measurements^8–10^. Non-binary analyses of serological data are present in the literature for a range of pathogens^10–18^ including influenza virus^19, 20^, but very few of these studies are able to look at non-vaccinated populations and none have the scale and precision presented here.

In the present study, we analyze a large set of general-population serum samples collected as residual serum from biochemistry and haemotology labs in four hospitals in southern Vietnam, from 2009 to 2013. Using a zero-inflated mixture modeling approach, we allow for up to seven serological states. To account for the large sample size in our model selection procedure, we use the Bayesian Information Criterion, and to avoid inference of spurious serological states we set additional criteria to ensure that inferred titer groups are epidemiologically meaningful. We hypothesized that serological classification of influenza antibody titers would be non-binary and that age and lineage exposure (H1N1 only) would be associated with certain titer groups. We found that H1N1 antibody titer distribution are best classified into four titer groups, that H3N2 is best classified into three groups, and that censoring may have prevented a complete classification of H3N2 titers.

## Results

**Figure 1:**
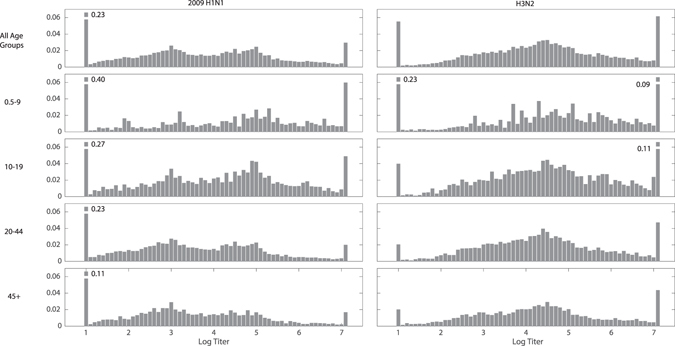
Antibody titer histograms for *n* = 20,152 individuals, plotted for all ages (top panels) and by age group (bottom four panels). Titers shown are to the HA1 components of the 2009 H1N1 pandemic influenza virus (left column) and to recently circulating H3N2 viruses (right column). The fractions of individuals with titers below the detection limit of 20 and above 1280 that were out of the plotting ranges are given next to the respective bar. Histograms were weighted to adjust for age and gender according to the Vietnam national housing census in 2009 for the four collection sites.

A total of 20,152 sera were collected and tested for antibody concentrations by protein microarray. The samples represent patients attending hospitals in four cities – Ho Chi Minh City (n = 5788), Nha Trang (n = 5630), Buon Ma Thuot (n = 4144), and Hue (n = 4590) in central and southern Vietnam. Titer distributions varied by age, as expected (Fig. 1) but did not vary by site (Figures S8 and S9). Figure 1 shows the age-stratified titer distributions to the HA1 component of the 2009 H1N1 virus and the most recently circulating H3N2 variants. If individuals truly represented seropositive (exposed) and seronegative (unexposed or naïve) categories, a mixture model of two components would classify samples into two subgroups. Visually, this does not appear to be the case as a broad range of titers was observed for both subtypes across all age groups. Thus, a mixture distribution fitting approach was employed to determine the appropriate number of components necessary to accurately describe the titer data.

**Figure 2:**
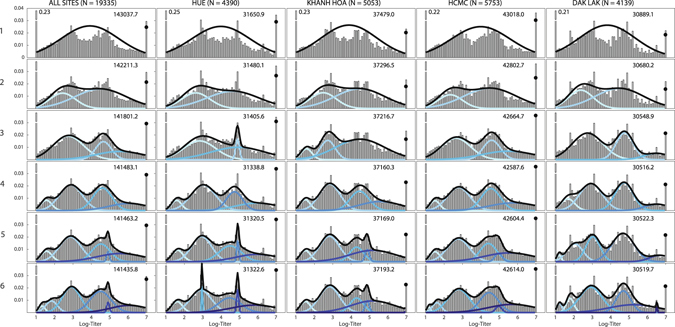
Titer histograms for 2009 H1N1, showing fit results for mixture models with different numbers of normal components (top to bottom; the label to the left of the *y*‐axis is the number of mixture components) and grouped by collection sites. Histograms are weighted to adjust for age and gender according to the Vietnam national housing census in 2009 for each of the four collection sites. The blue lines in each panel are the normalized probability density functions of the component distributions with darker colors used for increasing *μ*. The black lines show the full mixture distribution density, and the black dots are the estimated cumulative distribution of the mixture models at 7.0 (titer of 1280). The numbers in the upper right corner of each panel are the BIC scores of the model fits. The fractions of individuals with titers below the detection limit of 20 and above 1280 that were out of the plotting ranges were given next to their respective bars.

**Figure 3:**
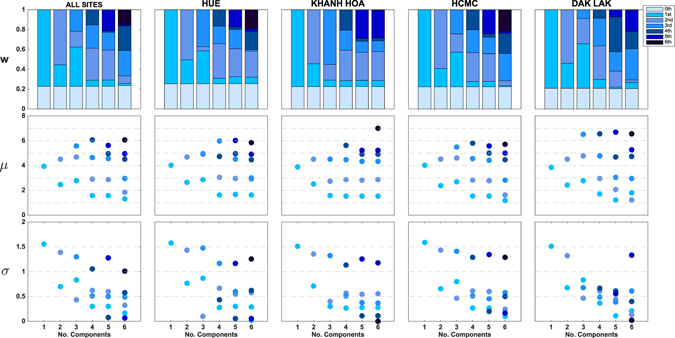
Visualization of model selection process for 2009 H1N1 titer-distribution models from Fig. 2. The *y*-axes show the fitted values of *w*_i_ (mixture weights), *μ*_i_ (means), and *σ*_i_ (standard deviations). Components’ shades were ranked from lightest to darkest in the order of increasing *μ*. In the top panel, the “0th component” represents the point mass *w*_0_ placed at 20 for titers below the lower detection limit of 20. Note that in many cases for five or six components, the weights or standard deviation parameters are close to zero; for some cases, two of the inferred mean parameters are very close to each other.

Mixture distribution fits for up to six components, with an additional weight at a log-titer of one (“zero inflation”), are shown in Fig. 2 for H1N1 and Fig. S10 for H3N2. For both subtypes, it is clear that a binary classification of titer is not the most informative interpretation of the titer distribution, as both the one- and two-component models (top two rows) did not capture the underlying structure of the dataset adequately. When stratifying the data by site (sample size >4,000), the Bayesian Information Criterion (BIC) selected four components as the best model for the H1N1 data (five for Hue, but the ΔBIC = 18 here was relatively small compared to other changes between nested models) and three components as the best model for H3N2. The five- and six-component models either overfit the data (according to the BIC) or included low-variance/low-weight components, which would correspond to an implausible population subgroup with a very specific antibody titer (Figs. 2 and 3). This was readily seen in the aggregate data which is why the BIC-selected models of the by-site data are likely to be better explanations of the structure of these titer distributions. BIC improvement from *n* mixture components to *n* + 1 components is shown in Table 1 for 2009 H1N1 and Table S7 for H3N2. The means and variances were allowed to be free in these analyses, and the confidence intervals for the inferred parameters (Appendix Section 7) suggest that the structure of the distributions and the inferred values were robust across the four sites in our analysis.

**Table 1:** Change in BIC values as the number of normal distributions in the mixture increases from one to six for 2009 H1N1, for the aggregated data as well as the individual collection sites.

		ALL SITES	HUE	KHANH HOA	HCMC	DAK LAK
		(N = 19335)	(N = 4390)	(N = 5053)	(N = 5753)	(N = 4139)
SumNegLLH, single component		71504.04	15812.88	18726.69	21496.01	15432.06
change in number of components	1 to 2	−826.40	−170.79	−182.44	−215.33	−208.86
	2 to 3	−410.07	−74.55	−79.83	−137.95	−131.34
	3 to 4	−318.12	−66.76	−56.37	−77.14	−32.68
	4 to 5	−19.87	−18.32	8.68	16.82	6.10
	5 to 6	−27.42	2.13	24.23	9.57	−2.59

The values of the negative sum of the log likelihood were weighted to adjust for age and gender according to the Vietnam national housing census in 2009. The first row shows the exact values of sum of the negative log-likelihoods (SumNegLLH).

The three- and four-component mixtures indicate that these data can be used to develop a more informative serological classification for influenza. Using known results for this microarray assay^3, 20, 21^, titers below 100 would be classified as negative or ‘not previously exposed to this particular influenza strain’. For H1N1, this indicates that titers in the first component *μ*_1_ = 29.8 (95% CI 29.1–30.5) and in the second component *μ*_2_ = 75.0 (95% CI 73.4–76.7) would both correspond to seronegative individuals. Similarly, for H3N2, seronegative individuals would be represented by the first component *μ*_1_ = 80.2 (95% CI 76.7–83.4). The second-highest titer component has mean *μ*_3_ = 247.3 (95% CI 240.8–261.7) for H1N1 and *μ*_2_ = 213.3 (95% CI 209.7–216.6) for H3N2. The highest titer component has mean *μ*_4_ = 670.9 (95% CI 519.8–787.9) for H1N1 and *μ*_3_ = 455.0 (95% CI 428.1–483.7) for H3N2. The natural interpretation of these high-titer subgroups – based on antibody titers measured as a function of time since infection^3^ – is that they represent more recent infections. As it is known that the influenza antibody decay rate is fast enough to be observed in the first six to twelve months after an acute infection^22, 23^, for H1N1 the highest titer subgroup may be an approximate designation for recently infected individuals, and the second highest titer subgroup may correspond to ‘historically infected’ individuals, i.e. individuals infected at some point in the non-recent past.

For H1N1, these interpretations are able to be validated using post-pandemic sera. Assuming that the highest-titer component (*w*_4_) of the mixture distribution corresponds to recently infected individuals and the second highest-titer component (*w*_3_) corresponds to historic infection, one would expect to be able to use the weights *w*_3_ and *w*_4_ as proxies for the pandemic attack rate. Looking at samples collected from January 2010 to June 2010 – i.e. after the first wave of the 2009 influenza pandemic in Vietnam^24, 25^ – the proportions of individuals that were recently infected with 2009 H1N1 were highest among younger individuals (0.14, 0.23, 0.08, and 0.16, for the 0.5–9, 10–19, 20–44, and ≥45 age groups, respectively), while the proportions of historically-infected individuals were approximately equal among age groups (0.16, 0.22, 0.23, and 0.20; same age groups); see Table S6 for confidence intervals. The estimates of 14% of children aged 0.5–9 and 23% of children aged 10–19 falling into the recently infected category are likely to be slight underestimates^5, 6, 24^ of pandemic attack rate as the post-pandemic sample here includes samples collected through June 2010. Nevertheless, these are within the expected ranges of the attack rate of the first year of the 2009 pandemic. For older individuals, pandemic attack rates are more difficult to validate but it is important to remember that older individuals had measurable antibody titers to 2009 H1N1 prior to the arrival of the new pandemic virus^9^. The pattern of attack rates observed in our samples is consistent with the two highest titer categories representing recent and historical infections with the H1N1 subtype.

The epidemiological interpretation of the H3N2 mixture components cannot be validated at present. The best-fit mixture models for H3N2 had larger variances than those for H1N1. The log-titer ranges (±2σ) for the three H3N2 titer groups were 26–240, 114–456, and 68–3045. Thus, the discriminatory power between the last two components was not as good as for H1N1 (see Fig. S10). The large standard deviations of the last component for H3N2 may have been the result of the high fraction of right-censored samples with titers ≥1280. In addition, the proportions of individuals in the highest titer group (third component) are 0.46 (ages 0.5–9), 0.49 (ages 10–19), 0.43 (ages 20–44), and 0.34 (≥45). These are unlikely to represent recent attack rates of H3N2 epidemics and are more likely to represent historical infection, i.e. individuals who have been exposed to the currently circulating H3N2 strain at some point in the past. One possible explanation for these observations is the existence of an additional fourth peak for the H3N2 titers describing individuals with titers above the upper limit of detection (≥1280). In our sample set, the proportions of individuals with H3 titers equal to 1280 were two to three times higher than those for H1N1 in the same age category. This is consistent with the existence of a fourth titer group with mean titer >1280, but we cannot confirm this with the current data as the samples were not diluted past 1:1280.

**Figure 4:**
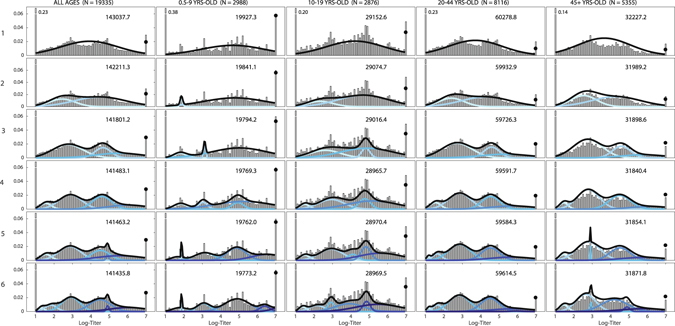
Titer histograms and fit results for mixture models with different numbers of components (label on the left is the number of mixture components) and grouped by different age groups recommended by the CONCISE (http://consise.tghn.org/) consortium for 2009 H1N1 influenza. Histograms are weighted to adjust for age and gender according to the Vietnam national housing census in 2009. The numbers in the upper right corner of each panel are the fitted BIC scores of the respective model. For each panel, the blue lines are the normalized probability density of the component distributions with darker colors used for increasing *μ*. Black lines are the total mixture distribution density; and the black dots are estimated probability weight of the mixture model for titers ≥7.0. The fractions of individuals with titers below the detection limit of 20 and above 1280 that were out of the plotting ranges are shown next to their respective bars.

For both subtypes, the individual components in the mixture models did not correspond to any specific age groups, and stratifying the samples by age did not explain any particular component of the mixture (Figs. 4, S12, and S15). All age groups included individuals with high, medium, and low titer levels. H1N1 has a more complex lineage history than H3N2, with three different lineages circulating since 1918. This suggests that separating the samples into H1N1 lineage-exposure groups (pre-1957, post-1977, post-2009) may account for certain titer groups or categories. However, separating the samples by birth year – 0.5–50 years-old and ≥60 years-old, to distinguish individuals that could and could not have been infected by the 1918-lineage H1N1 – did not provide any evidence for this effect (Fig. S14 and Table S9).

## Discussion

Using a large collection of serum samples and a continuous measurement of antibody titer, we were able to describe the natural distribution of antibody titers to the 2009 H1N1 and H3N2 subtypes of influenza virus. As there is almost no influenza vaccination in Vietnam and as influenza in Vietnam is characterized by a combination of local persistence and annual/biannual outbreaks^26–28^, characterization of titer distribution in this context is a useful general approach for looking at the immune status of a population at quasi-equilibrium with an endemic infectious disease. With a mixture model approach, we were able to identify the presence of multiple exposure groups in the population according to their titers. Our interpretation of these multiple exposure groups – according to titers measured for confirmed cases^3, 21^ and past measurements of the rate of antibody waning^22, 23^ – is that they represent recently infected individuals, historically (i.e. non-recently) infected individuals, and naïve individuals. Note that for influenza, a naïve individual is one who has not been exposed to the currently circulating strain, which means that there will be naïve individuals in all age groups.

This study used an atypical seroepidemiological design as the samples were collected continuously, and not specifically in a post-epidemic or post-pandemic scenario. In addition, the serum samples were collected in the tropics where continuous circulation of influenza virus is believed to occur^28–33^ and where populations are much less likely to be vaccinated for influenza (less than 0.8% annual coverage for Vietnam). Therefore, the present data set is the first to show the natural distribution of influenza antibody titers in a human population.

One useful application of these results in future serological studies is to encourage, by default, the inclusion of multiple serological states in the data analysis phase, which may result in a more informative classification of antibody titer than a separation into seropositive and seronegative. The classification proposed here uses antibody levels as proxies for recency of infection, and if correct, this should allow for a more informative reconstruction of the population’s epidemic history. In general, knowing the IgG antibody waning rate is essential for interpreting the titers measured in serological cross-sections^34, 35^, and using waning rates to estimate the time of past infection has already been attempted for some infectious diseases^36–39^, but not for influenza virus. Longitudinal follow-up studies that are able to provide accurate estimates of antibody waning rates are crucial for this type of analysis, but they are rare^3, 23, 40, 41^.

Two major limitations of serological classification systems will need to be better understood. First, a mixture distribution approach does not guarantee that individuals can be easily classified into one of several titer subgroups. With substantial overlap in some mixture components, individuals can have approximately equal probabilities of belonging to two or three different titer categories. In addition, individual variation will have a large effect on titer interpretations. A high-titer sample could represent a recent infection, but individuals can maintain high titers longer than the mean duration observed in clinical studies. This would normally, but not exclusively, be observed in children. Likewise, lower antibody titers (in the 200–250 range) could indicate historical past infection, a low response to a recent infection^42^, or a recent but mild infection. With serological data alone, these scenarios cannot be distinguished. For subtype H3N2 specifically, low titer levels could indicate cross-reactions between antibodies generated to an older influenza variant than the recent H3N2 HA1 proteins spotted on the protein microarray.

Second, a major challenge in influenza seroepidemiology is that it is difficult to take into account the effects of original antigen sin^42, 43^ or age-dependent seroconversion^40^ (ADS). Age-dependent seroconversion is distinct from original antigenic sin in that ADS assumes that individuals of different ages seroconvert to different titer levels irrespective of the individual’s infection history. In principle, the effect of ADS should be detectable for 2009 H1N1 infections in individuals younger than 50, as for these individuals an exposure to the 2009 virus would have been a first exposure. However, the mixture component means (*μ*_i_ parameters) and the component weights (*w*_i_) are not separately identifiable in the mixture model. Thus, we cannot state that the ‘recently infected’ titer subgroups are comparable across age groups, as the inferential process will make the exact definition of recency different for the 10–19 age group than for the 20–44 age group. Even if we were to assume that the fourth mixture components should be comparable across age groups, the titer means denoted by *μ*_4_ in Fig. 4 do differ but are within one standard deviation of one another. Thus, there is a lack of evidence for ADS in our titer data. As we only considered recent antigens in this analysis, effects of original antigenic sin were not able to be investigated.

The next critical step in this analysis will be using titer data from follow-up on confirmed cases^3^ to determine if the natural distribution of antibody titers conforms to the recent, historical, and naïve categories as presented here. If antibody waning rates can be measured with a high degree of precision, these may allow for a detailed description of individuals’ recency of infection and possibly a reconstruction of past epidemic history in human populations. Large-scale serological studies like the one presented here are labor-intensive and slow to generate results. Nevertheless, the long follow-up and the large sample size will be worth it if seroepidemiology can be pushed forward to maximize the amount of biological information that can be extracted from population-level serology studies.

## Materials and Methods

Residual serum samples were collected from four hospital laboratories in southern Vietnam: the Hospital for Tropical Diseases in Ho Chi Minh City (urban, densely populated), Khanh Hoa Provincial Hospital in Nha Trang city (small urban, central coast), Dak Lak Provincial Hospital in Buon Ma Thuot city (central highlands, rural), and Hue Central hospital in Hue City (small urban, central coast). Samples were collected from July 2009 to December 2013 on a bimonthly basis; 200 were included in each collection from all age groups (neonates to elderly individuals in their 90s). Samples were anonymized, delinked, and labeled with age, gender, originating hospital ward (HIV wards were excluded), and date of collection. Samples were collected from both inpatients and outpatients and are believed to represent the hospital-going population in their respective cities. This assumption is currently being tested and will continue to be tested as different antibody assays are performed on the sample set. Two early analyses (one unpublished and one published^44^) suggest that when looking at hospital presentation with hepatitis, the younger age range (<20) in the sample set may represent a sub-population more vulnerable to infectious disease exposure than the general population. The sample collection described here is part of a large ongoing study in serial seroepidemiology^1, 2, 34^ aimed at describing the dynamics of influenza circulation in southern Vietnam. The study was approved by the Scientific and Ethical Committee of the Hospital for Tropical Diseases in Ho Chi Minh City and the Oxford Tropical Research Ethics Committee at the University of Oxford.

The samples were tested for presence of influenza antibodies using a protein-microarray (PA) method^45^, at serial four-fold dilutions from 20 to 1280, to test for IgG antibody to the HA1 component of 16 different influenza viruses^1^. Two-fold dilutions were used in some instances; see validation of this approach in Appendix Section 2. A sample of the international standard (IS) for testing antibody response to influenza A H1N1 Pandemic 2009 (H1-09) was included on every slide to correct for inter-laboratory, inter-technician, and inter-slide variations^45^ (Appendix Section 1.2). Assay repeatability was assessed using a positive control and replicates of patient samples (Appendix Section 3). Titers were defined as the dilution at which samples yield a median response between the minimum and maximum luminescence values of 3000 and 65535. Titers of all human samples on each slide are normalized based on the IS titers of the reference antigen against its geometric mean (Table S2). In this analysis, titers to the 2009 H1N1 virus (A/California/6/2009) and recently circulating H3N2 viruses (geometric mean titer to A/Victoria/210/2009 and A/Victoria/361/2011) were analyzed.

To describe the distribution of influenza antibody titers in the Vietnamese population, titer values were separated by site, adjusted to their province’s age and gender distribution^46^ (Appendix Section 4), and plotted as a simple weighted histogram (Fig. 1). A series of mixture models was used to fit this distribution, with the assumption being that individual samples have one of several immune statuses which are represented by the different components in the mixture model. Our hypothesis was that the sample population consists of different subpopulations with different antibody levels depending on their infection history and that each of these components can be represented by a single parametric distribution.

Titers were log-transformed and assumed to come from a *C*-component mixture distribution with the corresponding likelihood:

1 $$ {\mathcal L} ({\rm{x}}|\,{\boldsymbol{\theta }}\,)=\,\prod _{i=1}^{n}\,\sum _{j=1}^{C}{w}_{j}\cdot {f}_{j}(\,{{\rm{x}}}_{i}\,|\,{\theta }_{j}\,)$$

where *f* is the probability density function of a normal distribution with parameters *θ*_*j*_ and **w** = (*w*_1_, *w*_2_, …, *w*_C_) is the vector of component weights in the mixture. The log-likelihood was defined as:

2 $$\ell ({\rm{x}}|{\boldsymbol{\theta }}\,)=\,\sum _{i=1}^{{\boldsymbol{n}}}{s}_{i}\cdot \,\mathrm{log}[\sum _{j=1}^{C}{w}_{j}\cdot {f}_{j}(\,{{\rm{x}}}_{i}\,|\,{\theta }_{j}\,)]$$

in which the *s*_*i*_ parameters are sampling corrections to adjust the sample age and sex distribution to the population’s true demographic distribution; *f*_*j*_(*x*_*i*_|*θ*_*j*_), j = 1, 2, .., *C* is the probability density function that a given sample *x*_*i*_ belongs to the *j*th-component in the mixture. *C* is the number of mixture components^47, 48^.

The microarray assay produces continuous log-titer results between 1.0 (titer of 20) and 7.0 (titer of 1280). To account for these detection limits, an extra probability weight *w*_0_ was added at 20 to account for samples that had antibody concentrations at or below the detection limit of 20. This can be considered a zero-inflated mixture model, where titers of 20 are the “zeroes”. Because of this added probability mass, we discretized the probability mass functions to make the entire distribution discrete; hence the distributions *f* formally represents discretized versions of continuous density functions (Appendix Section 5). At the upper detection limit of 7.0, the mixture distribution was censored assuming that individuals with titers of 7.0 represented a class of seropositive individuals with a real titer value if the assays had been continued to be diluted until the real titer was found. Censoring on the right and truncating on the left gave the best fit (according to BIC) among the four combinations. Truncating on the left means that the extra weight on the left-hand side of the probability density function (the portion below 20) was simply discarded when performing the fits, as “zero-inflation” on the left-hand side was used to fit the number of samples that had titers of 20 or below.

Thus, the log-likelihood in (2) was modified as:

3 $${\ell }^{interval}({\rm{x}}|\,{\boldsymbol{\theta }}\,)=\,\sum _{i=1}^{{\boldsymbol{n}}}{s}_{i}\cdot \,\mathrm{log}[{w}_{0}\,+\,\sum _{j=1}^{C}{w}_{j}\cdot {f}_{j}(\,{{\rm{x}}}_{i}\,|\,{\theta }_{j})]$$

Maximum likelihood estimation was carried out using the Nelder-Meade algorithm implemented in Java 8.0 (Apache Commons Math 3.3). Global optima and convergence were assessed by starting searches from different sets of the initial conditions. Weibull, Gamma, and normal distributional forms were tested for the mixture components, and as there was little difference in the fits (Appendix Section 6), normal distributions were chosen for the analysis. Confidence intervals for means, variances, and weight parameters *w*_*j*_ were computed using likelihood profiles^48^.

For multi-component mixture models, the likelihood ratio test between a specific model and its immediate predecessor (e.g. *n* components versus *n*-1 components) is not a valid statistical comparison. Since interchanging the components’ identity gives the same mixture likelihood^48^, the regularity conditions do not hold for the likelihood ratio test to have its usual χ^2^ distribution. Thus, the most appropriate number of mixture components was chosen by (1) Bayesian Information Criterion to take into account the number of samples, and (2) a qualitative inspection of the means and variances of the components to ensure that (*a*) multiple means did not overlap and (*b*) variances and weights were not too small, which would make them not epidemiologically meaningful.

Data on influenza vaccine imports in Vietnam were obtained from Vietnam’s Customs and Imports Department via IMS Health Vietnam. Annual influenza vaccine imports for 2014–2016 are sufficient to cover approximately 0.8% of the Vietnamese population. As wealth and access to medicines are growing in Vietnam, the coverage level for 2009 to 2013 was likely to be lower than 0.8%.

### Data Availability Statement

Data are available from the authors upon request.

## Electronic supplementary material

Supplementary Appendix
