# Supplementary material for: Structure of general-population antibody titer distributions to influenza A virus
Source: Sci Rep. 2017 Jul 20;7:6060. doi: 10.1038/s41598-017-06177-0 (PMC5519701; doi:10.1038/s41598-017-06177-0)
Supplement: Supplementary file 1 — Supplementary Appendix [file 41598_2017_6177_MOESM1_ESM.pdf]

Supplementary Online Appendix

Structure of general-population antibody titer distributions to influenza A virus

Nguyen Thi Duy Nhat, Stacy Todd, Erwin de Bruin, Tran Thi Nhu Thao, Nguyen Ha Thao Vy, Tran Minh Quan, Dao Nguyen Vinh, Janko van Beek, Pham Hong Anh, Ha Minh Lam, Nguyen Thanh Hung, Nguyen Thi Le Thanh, Huynh Le Anh Huy, Vo Thi Hong Ha, Stephen Baker, Guy E Thwaites, Nguyen Thi Nam Lien, Tran Thi Kim Hong, Jeremy Farrar, Cameron P Simmons, Nguyen Van Vinh Chau, Marion Koopmans, Maciej F Boni

---

Table of Contents

1. Mapping luminescence values to titers using luminescence values at four dilutions
   1.1 Four-parameter log-logistic curve
   1.2 Standardization against the international Influenza A H1N1 Pdm 2009 Positive Control
2. Mapping luminescence values to titers using luminescence values at two dilutions
3. Titer reproducibility
4. Representativeness of sample collection versus census data
5. Choosing bin width for discretizing the normal distribution
6. Comparisons of gamma, Weibull, and normal fits
7. Confidence intervals for mixture component estimates
8. Confidence intervals for pandemic attack rate
9. Additional figures and tables

1. Mapping luminescence values to titers using luminescence values at four dilutions

Samples were tested for presence of a range of seasonal and pandemic influenza antibodies using the protein-microarray (PA) method at serial four-fold dilutions from 20 to 1280 except for the international standard (IS) positive control against H1N1 Pandemic 2009 that was tested from 40 to 2560. Commercially available HA1 proteins of the 16 influenza viruses (Table S1) were spotted in duplicates on each of the 64-pad nitrocellulose coated slide as described by Koopmans et al (*Clinical Microbiology and Infection*, 2012, 18(8):797-807). All of the slides used in this study were printed at the Erasmus Medical Center (Rotterdam, NL) and the Dutch National Institute for Public Health and Environment (RIVM; Bilthoven, NL) by Erwin de Bruin. On each slide, 15 human sera and one positive control were tested in BSL-2 conditions at the Oxford University Clinical Research Unit (OUCRU) in Ho Chi Minh City, in the same manner described by Koopmans et al (2012). The processed slides were then dried and sent back to RIVM for scanning. The antigen signals were quantified by a Scanarray Gx Plus microarray scanner (PerkinElmer) with an adaptive circle size of 80−200μm. Digital copies of the scan images were analyzed using ScanArray Express (version 4.0) Software (PerkinElmer) to determine the median fluorescence intensity. The PA titer was defined as the best fit titer according to a four-parameter log-logistic function described in the next section. Fitted titers were then standardized to a positive control in order to correct for variation across slides; values falling outside the [20, 1280] range were scored as 10 and 1810. All data analysis was done in Matlab R2015a. The initial script was written by Janko van Beek.

Table S1: List of all influenza strains tested on the microarrays

Human Antigens		Abbreviation	Avian Antigens		Abbreviation
**H1N1**	A/South Carolina/1/18	H1-18	**H5N1**	A/Vietnam/1194/2005	H5-05
	A/USSR/92/1977	H1-77		A/Cambodia/R0405050/2007	H5-07
	A/New Caledonia/20/1999	H1-99		A/Hubei/1/2010	H5-10
	A/Brisbane/59/2007	H1-07	**H7N7**	A/Chicken/Netherlands/2003	H7-03
	A/California/6/2009	H1-09	**H9N2**	A/GuineaFowl/HK/1999	H9-99
**H3N2**	A/Aichi/2/68	H3-68			
	A/Wyoming/3/2003	H3-03			
	A/Wisconsin/67/2005	H3-05			
	A/Brisbane/10/2007	H3-07			
	A/Victoria/210/2009	H3-09			
	A/Victoria/361/2011	H3-11			

1.1 Four parameter log-logistic curve

For each human-antigen combination, the eight luminescence scores obtained from serial four-fold dilutions were fitted, using non-linear least-squares regression, to a four-parameter log-logistic curve (Figure S1); errors were defined as absolute luminescence differences between the model and the data point. The log-logistic luminescence curve is sigmoidal in shape. The maximum readout given by the scanner was 65355; thus, 65356 was used as the maximum luminescence in the log-logistic fit. The minimum read out was defined as 3000, below which it was difficult to differentiate between the antigen signals and the background luminescence. Mathematically, the titer value was determined as the *x*-value of the inflection point of the fitted log-logistic curve, which is equivalent to the point where the fitted curve achieves a luminescence of 34268. Samples were assigned titer values of NaN if the width of the 95% confidence interval resulting from the fit was greater than one log2-unit. The variables *titer* and *b* were fit with 8 luminescence data points using the equation below:

$$\mathrm{Lum}_i = c + \frac{d - c}{1 + \exp\left(b \cdot \log\left(\frac{\mathrm{dilution}_i}{\mathrm{titer}}\right)\right)}$$

in which: Lum_i : luminescence score at dilution *i*
c = 3000: minimum luminescence
d = 65535: maximum luminesce
titer: dilution of the inflection point

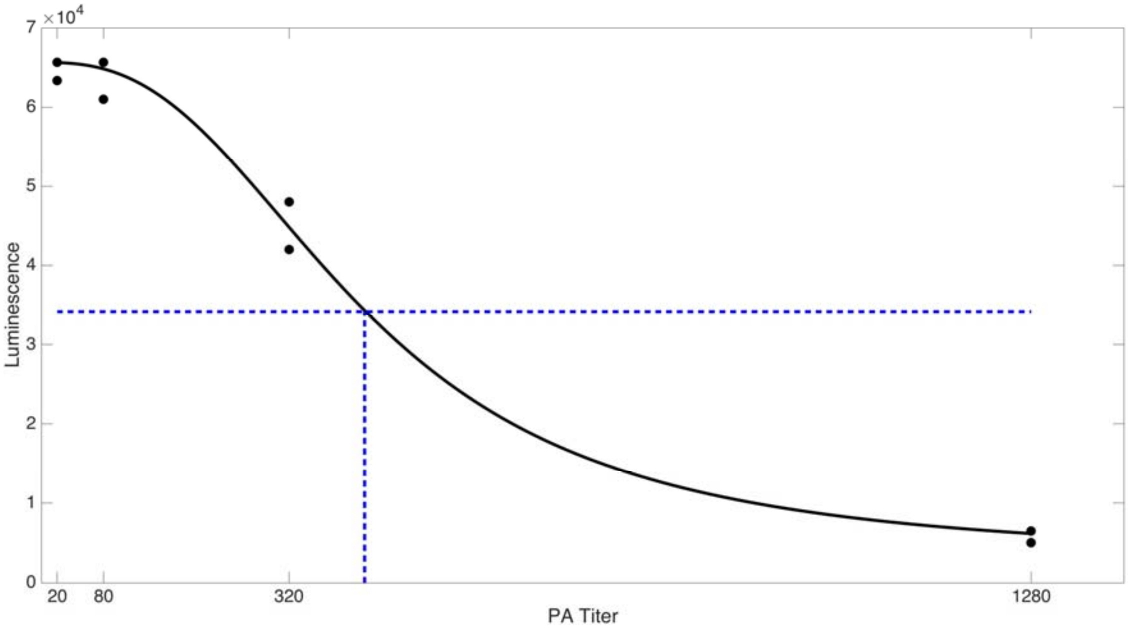

Figure S1: Luminescence vs. protein micro-array (PA) titers being fitted to four-parameter log-logistic curve in which c = 3000, d = 65536, titer = dilution at the inflection point, and b = slope at the inflection point

1.2 Standardization against the international positive control

A sample of the WHO 2nd International Standard (ISH2) for antibody to influenza H1N1pdm virus (H1-09) (National Institute for Biological Standards and Controls (NIBSC), NIBSC code: 10/202) was included on every slide to correct for variation among slides, technicians, slide batches (different printing runs), and slide series (different commercial antigen purchases). In order to take into account the titer change across series, the standardization procedure was divided into a two-step procedure. First, all samples within the same slide series were corrected to the geometric mean titers for ISH2 samples of a reference antigen within the respective series. Second, depending on the difference between the geometric mean of the reference antigen for series *s* compared to its geometric mean in series 1, the titers were deflated/inflated over the geometric-mean titers of series 1. In our analysis, the pandemic 2009 H1N1 (H1-09) antigen was chosen as the reference antigen for correcting variance across slides due to its stable performance over time (Table S2).

Table S2: Summary of ISH2 performance against all antigens

Antigen	Series 1		Series 2				Series 3			
	Mean	Std	Mean	Std	Inflation over Series1	P-value	Mean	Std	Inflation over Series1	P-value
H1-18	144.15	59.04	250.23	91.47	1.74	0	149.35	48.56	1.04	0.76675
H1-77	762.14	388.88	1091	514.66	1.43	0	905.99	312.2	1.19	0.00031
H1-99	287	156.21	307.74	121.12	1.07	0.01314	190.88	39.08	0.67	0.00491
H1-07	247	110.69	325.42	119.93	1.32	0	483.18	179.65	1.96	0
H1-09	158.91	44.76	154.15	89.66	0.97	0.48132	201.72	54.78	1.27	0
H3-68	348.95	145.17	542.08	103.93	1.55	0	458.56	87.03	1.31	0.00064
H3-03	392.81	119.55	550.42	275.15	1.40	0	448.03	94.08	1.14	0
H3-05	679.28	336.05	631.39	277.52	0.93	0.00109	777.45	209.43	1.14	0.01081
H3-07	126.14	37.55	189.85	75.41	1.51	0	158.85	93.98	1.26	0
H3-09	108.86	40.07	159.54	56.99	1.47	0	171	65.97	1.57	0
H3-11	146.08	61.06	214.03	82.15	1.47	0	174.75	64.58	1.20	0
H5-04	40.01	0.23	40.27	12.36	1.01	0.21794	NaN	NaN	NaN	NaN
H5-07	40.03	0.52	41.14	19.01	1.03	0.02606	NaN	NaN	NaN	NaN
H5-10	40.01	0.18	40.34	5.34	1.01	0.03549	40.33	24.04	1.01	0.21348
H7-03	40.23	14.71	40.96	48.39	1.02	0.36462	40.22	2.85	1.00	0.83947
H9-99	47.28	27.6	89.58	35.03	1.89	0	69.7	25.59	1.47	0

Mathematically, the standardization procedure looks as follows. The within-series correction factor or normalization factor for sample *s* on slide *i* and series *k* is calculated as:

$$CF_{ik} = \frac{GMT_{ISH1,H109,k}}{T_{ISH1,i,H109,k}}$$

in which $T_{ISH1,i,H109}$ is the H1-09 titer of the positive control on slide *i* (assuming it is in series *k*); and $GMT_{ISH1,H109,k}$ is the geometric mean of ISH2 titers for the H1-09 antigen in series *k*. Hence, the normalization factor is the same for all antigens a and all samples s on the same slide; and it serves as the rejection criterion to discard slides whose H1-09 titers of the positive control differ by more than 1.2 dilutions (log2-units) away from $GMT_{ISH1,H109,k}$.

To correct for the inter-series variation, the mean titer of antigen a in series k is inflated/deflated over its corresponding mean from series 1:

$$CF'_{ak} = \frac{GMT_{ISH1,a,1}}{GMT_{ISH1,a,k}}$$

The individual titer of antigen a for all samples s on slide *i* in series *k* is then corrected as:

$$Titer_{saik} = CF_{ik} \cdot CF'_{ak} \cdot Fitted\_Titer_{saik}$$

2. Mapping luminescence values to titers using luminescence values at two dilutions

After analyzing about 19,000 human sera at four-fold dilutions (~300,000 sample-antigen combinations), we began a process of mapping luminescence series to titers to determine if fewer luminescence points could be used for titer inference. This was done to reduce the number of dilutions done per sample in order to reduce cost.

Figure S2 shows the relationship between luminescence scores at single dilutions and the titer obtained from a four-fold dilution. Clearly, a single dilution is not sufficient to be used as a proxy for a four-fold titer, as the majority of mappings from single dilutions to titers would result in mapped titers that are one or two log units distant from the true titer. The single dilution at 20 is especially uninformative, as 80% of the luminescence scores at dilution 1:20 are saturated at 65535 (Figure S2, top-left panel). Thus, we considered three dilution pairs as the best candidates for mapping luminescence pairs to true titers. The three pairs considered were 1:80 and 1:320, 1:80 and 1:1280, and 1:320 and 1:1280. The objective was to define a mapping function $f(L_1, L_2) = T$, where T is the true titer obtained from four-fold dilutions and L_1 and L_2 are luminescence scores at two different dilutions. Four different functional forms of f were investigated:

1. a simple grid approach where the $L_1 \times L_2$ space was divided into grid squares of size $n \times n$ luminescence points on each side. Values of n = 100, 200, 500, and 1000 were tried. The exact $L_1 \times L_2$ space used was [3000, 65536] × [3000, 65536]. The median titer was used as the mapped titer point in each grid square. This function gave poor mapping power at the two ends, titers below 200 and above 1000. This quality of the mapping did not improve with smaller grid size.

2. a piecewise linear function $f(L_1, L_2) = \begin{cases} aL_1 + bL_2 & for L_1 < 65355 \; and \; L_2 \le L_1 \\ c + dL_2 & for L_1 = 65355 \; and \; L_2 \le L_1 \end{cases}$ with the breaks occurring at the luminescence points where the titers achieve their boundary values; i.e. titer of 1810 was assigned for $L_2 > 34268$.

3. a linear spline surface plot of $f(L_1, L_2)$ using a linear interpolation method with the MATLAB family of functions `fitobject`.

4. a cubic spline surface plot of $f(L_1, L_2)$ using cubic spline interpolation method with the MATLAB family of functions `fitobject`.

Functional form 4 was chosen as it had the best accuracy across all titer ranges above a titer of 150, although functional form 3 had similar accuracy. None of the methods were able to accurately map to titers below 150 when the smallest dilution used was 1:320.

However, the overall performance of the mapping was considerably poorer using the 1:80/1:320 and 1:80/1:1280 pairs which is why 1:320 and 1:1280 were chosen as the two most informative dilutions to use for inferring titers (see below).

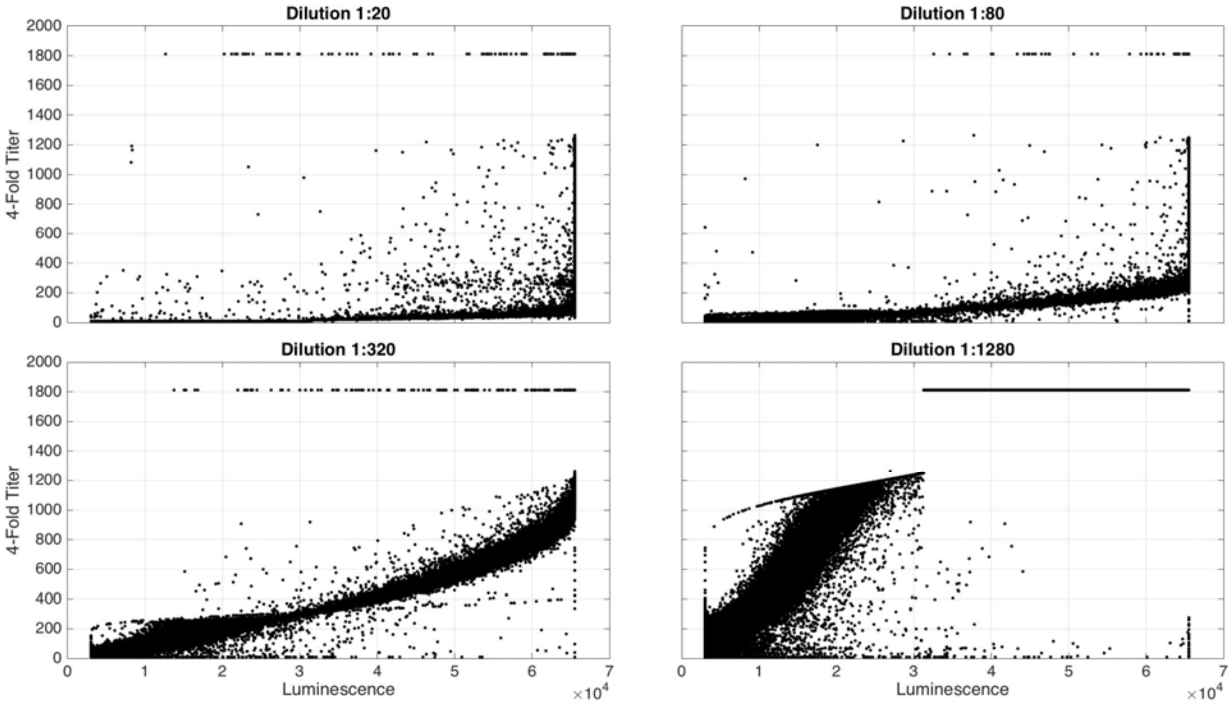

Figure S2: Four-fold titer vs. luminescence scores at each of the four dilutions from 1:20 to 1:1280

Figure S3 shows the mapped 2-fold titers using functional form 4 against the true 4-fold titers. Three different dilution pairs for the 2-fold titers are shown in the three panels. The 1:80 and 1:320 dilution pair lost discriminatory power for titer values above 700. The 1:80 and 1:1280 dilution pair, on the other hand, failed to map titers in the intermediate range [400, 1000]. Even though the 1:320 and 1:1280 dilution pair did not map titers below 200 accurately, this should not affect the seroprevalence estimates because the mean of the highest titer component in the main text are higher than 200 for all age-groups (Table 1, main text). In other words, losing discriminatory power among the seronegative groups should not affect seroprevalence estimates. After the initial 19,000 sera were processed using 4-fold titers, subsequent sera were processed at dilutions 1:320 and 1:1280 only (with the exception of Ho Chi Minh City samples, which were always processed 4-fold). To ensure accurate standardization across slides, the positive control samples (ISH2) were always processed at four dilutions; and the standardization procedure presented in section 1.2 was unchanged.

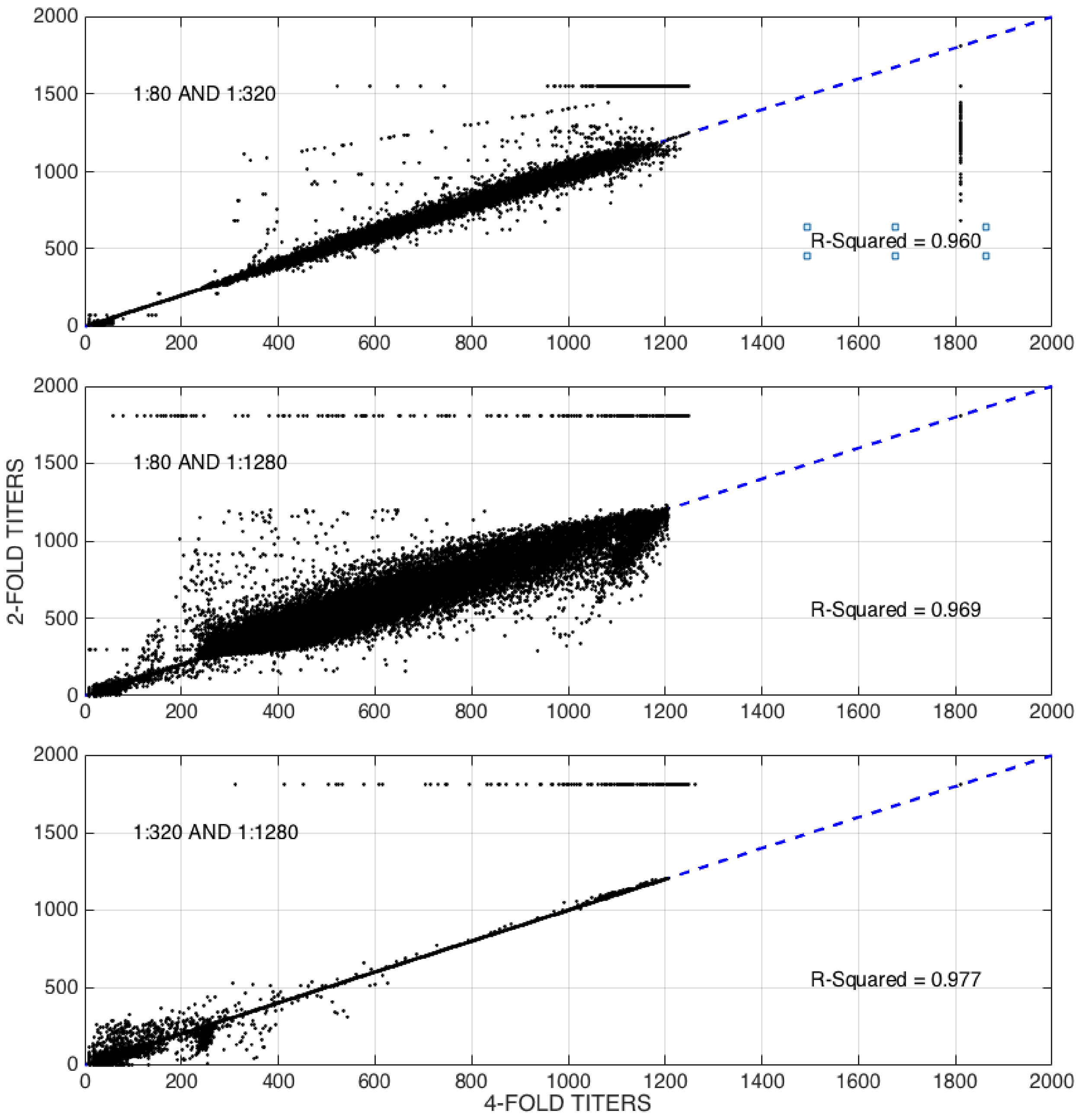

Figure S3: 2-fold titer obtained from cubic spline surface map vs. 4-fold titer for different pairs of dilutions 1:80 and 1:320, 1:80 and 1:1280, and 1:320 and 1:1280 (top down)

3. Titer Reproducibility

Thirty-two samples collected from Khanh Hoa were tested 11 times by different lab technicians on different days to assess titer reproducibility. Across all sixteen antigens on the array, 98% of the sample-antigen combinations had a standard deviation of log-titer less than one when performing replicate experiments (Figure S4, top panel), making PA titers more reproducible compared to standard HI assays.

We also looked at the reproducibility of the positive control (ISH2); see Figure S4, bottom panel. The median difference between an individual ISH2 titer and the geometric mean of all (n = 1457) ISH2 titers was below 0.5.

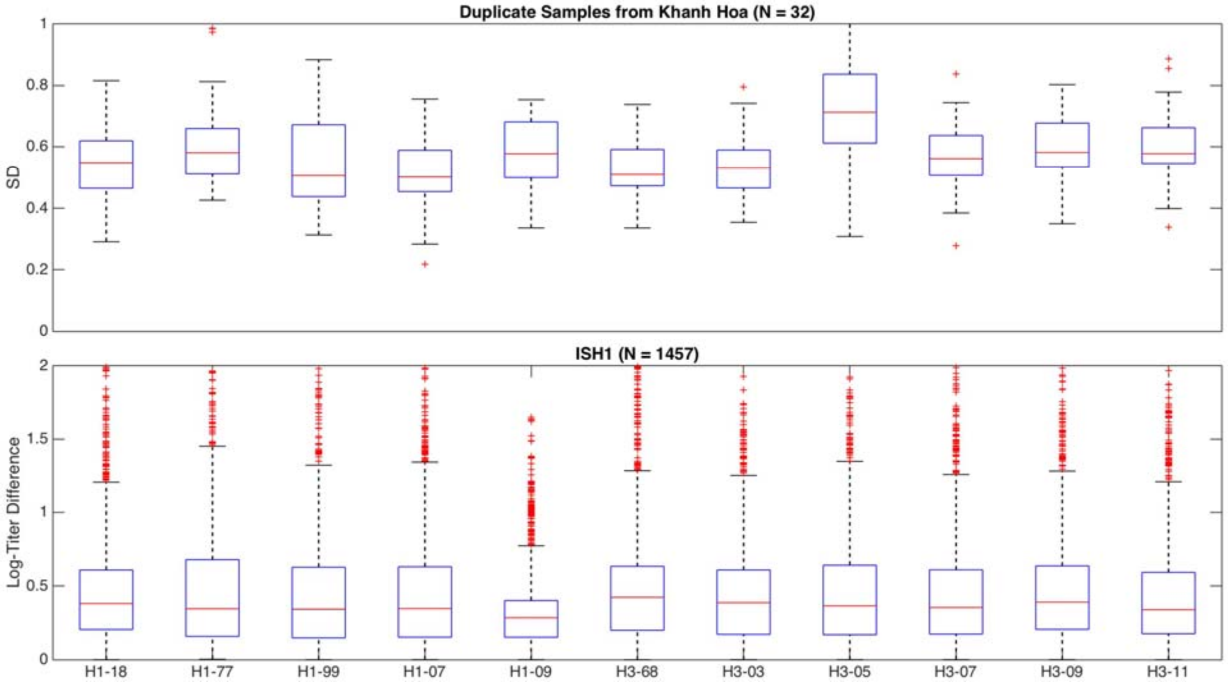

Figure S4: **Top:** Box plots of standard deviations of log2-titers for eleven human antigens (*x*-axis) for eleven replicates of 32 different samples from Khanh Hoa. In other words, each box plot summarizes 32 standard deviations. **Bottom:** Differences between individual ISH2 titers and the geometric mean of the ISH2 for 1457 replicates on 11 human antigens (log titer scale). In both plots, the red line is the median difference; and top and bottom edges are 75th and 25th percentile for each box, respectively. The whiskers extend to $q_3 + 1.5*(q_3 - q_1)$ and to $q_1 - 1.5(q_3 - q_1)$, where q_1 and q_3 are the 25th and 75th percentiles. Outliers are plotted in red dots.

4. Representativeness of sample collection versus census data

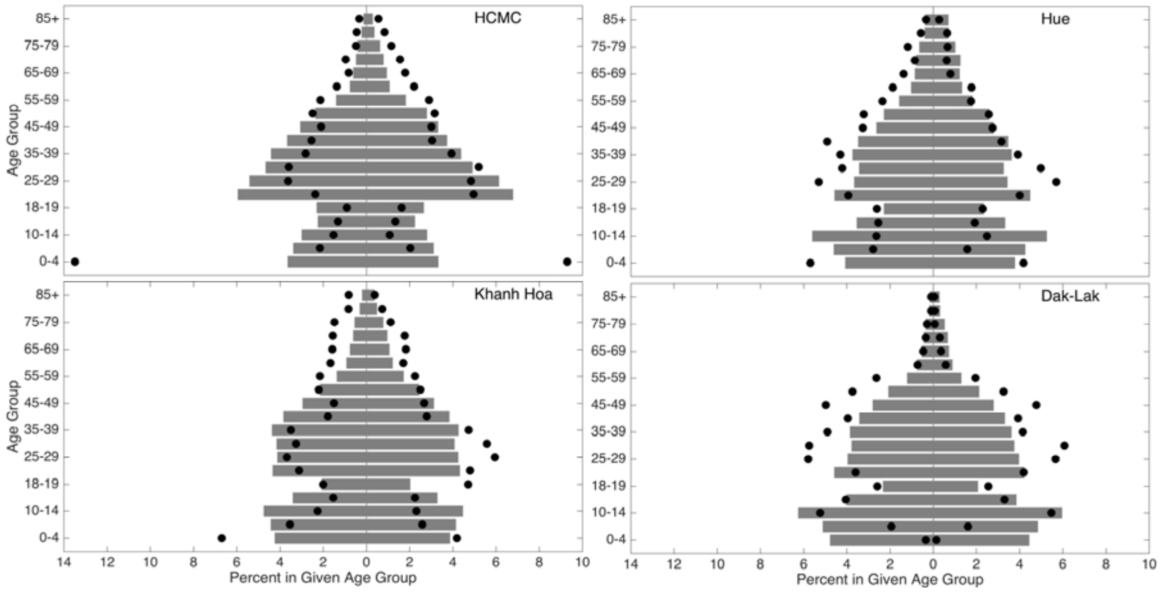

Figure S5: Collected sample population (black dots) versus Vietnam national housing census in 2009 (gray bars) for different sites. The *y*-axis shows different age groups and *x*-axis is the percentage of the population or sample in that age group. Left columns are data for males and right columns for females.

The 0-4 yrs-old age group was clearly oversampled for Ho Chi Minh City, and undersampled for Dak Lak, which might have been caused by different health care systems in these two regions causing pediatric admissions to be over/under-represented in certain hospitals. Ho Chi Minh City is a densely populated city with easy access to many levels of healthcare, whereas Dak Lak Provincial Hospital is in a small city in a rural region of the Vietnamese highlands, with a lower socioeconomic level and less access to healthcare. For some of the age groups, the sample was very close to that of the data (older than 40, both genders, Hue; older than 50, both genders, Ho Chi Minh City and Khanh Hoa; older than 60, both genders, Dak-Lak). For the 5-20 age group, all of the sites are undersampled for both genders, which is to be expected as these individuals are the least likely to report to hospitals. Overall, the serum sample set had an age structure similar to the true demography; thus, the sample weights included in the likelihood function introduced only minor adjustments.

The sample weights s_i in the likelihood equations were calculated from these data. For example, the fraction of males in the 0-5 year-old age range in the census data for HCMC is 0.0364 while this fraction is 0.1241 for the sample set. Thus, every sample that was male

and belonged to the 0-5 age category in the sample should be down-weighted by a factor of s_i = 0.0364 / 0.1241= 0.2933.

5. Choosing a bin width for discretizing the normal distribution

When computing the likelihood function, the normal distribution needed to be discretized so that a 'zero-inflated' normal distribution could be constructed (log-titer = 1 was the inflated probability mass). A probability mass and continuous probability function cannot be combined into a single likelihood function.

The normal mixture was defined on the log-titer range [1, 7], and seven different bin widths of 1.000, 0.500, 0.100, 0.050, 0.010. 0.005, and 0.001 were tested in this range to investigate the behavior of the seven different probability mass functions in the likelihood optimization algorithm (Table S3). For a single normal distribution fit, the optimum was the same for all bin widths. In the case of two and three components in a mixture distribution, there were few changes in the fitting results, unless the bin width was less than or equal to 0.100. It also was important to avoid using too small of a binwidth to avoid the problem of having "a spike" in the fitted distribution that had no epidemiological meaning. A binwidth of 0.100 was used in the analysis.

Table S3: LLH optimization results for different bin widths

Interval Width	w_0	w_1	μ_1	σ_1	w_2	μ_2	σ_2	w_3	μ_3	σ_3	SumNegLLH
1.000	0.228	0.772	3.945	1.551							38357.07
0.500	0.228	0.772	3.946	1.551							48240.31
0.100	0.228	0.772	3.950	1.551							71504.04
0.050	0.228	0.772	3.950	1.551							81535.65
0.010	0.228	0.772	3.949	1.551							104838.69
0.005	0.228	0.772	3.949	1.551							114876.59
0.001	0.228	0.772	3.949	1.551							138181.93
1.000	0.228	0.150	2.063	0.234	0.622	4.387	1.386				37870.75
0.500	0.228	0.211	2.443	0.686	0.561	4.509	1.398				47830.31
0.100	0.228	0.218	2.462	0.698	0.555	4.532	1.393				71076.04
0.050	0.228	0.218	2.462	0.697	0.555	4.532	1.392				81105.93
0.010	0.228	0.218	2.462	0.697	0.555	4.531	1.393				104410.41
0.005	0.228	0.218	2.462	0.697	0.555	4.532	1.393				114448.07
0.001	0.228	0.218	2.462	0.697	0.555	4.532	1.393				137753.27
1.000	0.228	0.205	2.017	0.059	0.377	4.026	0.794	0.190	5.765	1.207	37634.10
0.500	0.228	0.379	2.735	0.812	0.151	4.640	0.426	0.242	5.422	1.352	47639.60
0.100	0.228	0.396	2.771	0.830	0.159	4.684	0.434	0.217	5.571	1.298	70856.20
0.050	0.228	0.398	2.774	0.830	0.161	4.685	0.433	0.214	5.594	1.288	80881.55
0.010	0.228	0.398	2.774	0.830	0.161	4.684	0.434	0.214	5.591	1.290	104186.77
0.005	0.228	0.398	2.774	0.830	0.161	4.684	0.435	0.213	5.597	1.287	114224.69
0.001	0.228	0.398	2.774	0.830	0.161	4.684	0.435	0.213	5.596	1.288	137529.83

6. Comparisons of gamma, Weibull, and normal fits

Normal distributions were chosen for the form of the mixture components, as this is the traditional distributional form chosen for mixture distribution fits of continuous quantities when no other information is known or assumed about the data points. Comparisons were done with mixtures of Weibull and gamma distributions, although note that both Weibull and gamma distributions allow for symmetry and right skew, but not for left skew. In choosing a distributional form, when all other considerations are equal, a normal distribution form should be chosen over Weibull or gamma forms to prevent fits where rights skews are "discovered" by the fitting process but left skews remain undiscovered.

In the figures below, the four-component fits for H1N1 have the lowest BIC value when normal distributions are used as components. For three-component fits for H3N2, the gamma-fits have a somewhat lower BIC value than the normal fits (ΔBIC≈19), but it is also clear that the component structure in this fit does not distinguish titer groups. Normal components had better BIC than Weibull components for both H1N1 and H3N2.

Based on (1) our initial considerations of skewness in the fits, (2) the BIC values when comparing the fits for both subtypes, and (3) our visual inspection of the fits, we concluded that we did not have any evidential support for using gamma distributions or Weibull distributions in the mixture analysis.

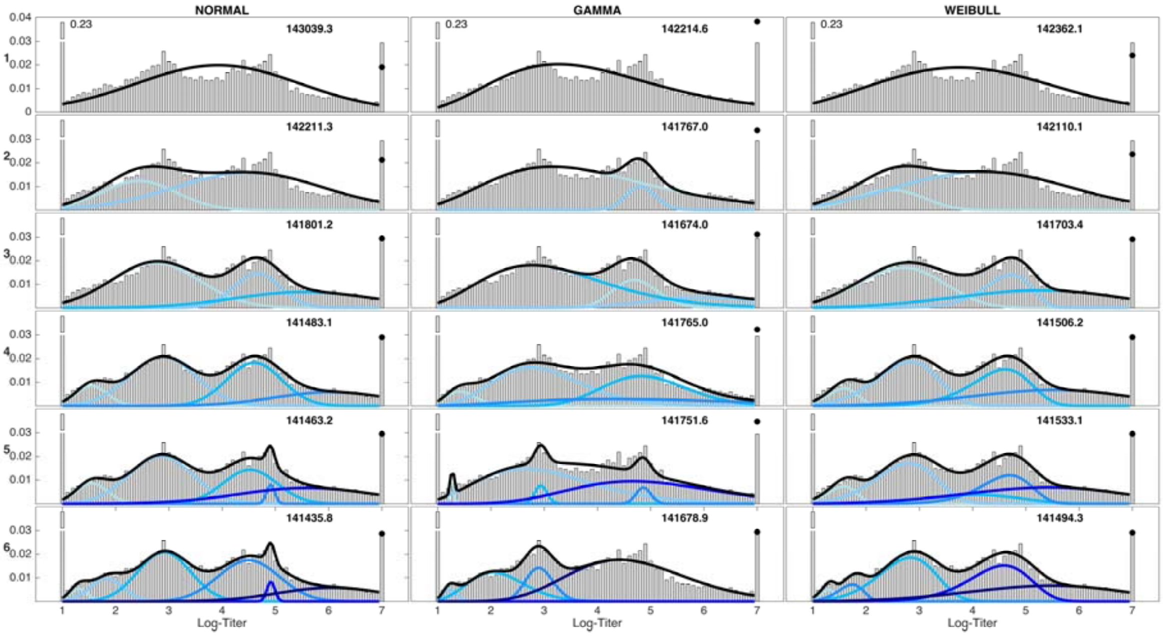

Figure S6: H1N1 titers, all sites all ages. Mixture fits were performed with three different distributional forms, shown in the three columns. The first row of figures shows fits with a single mixture component, the second row of figures shows fits with two mixture components, etc. The number in the top-right corner of each panel is the BIC value for that model fit.

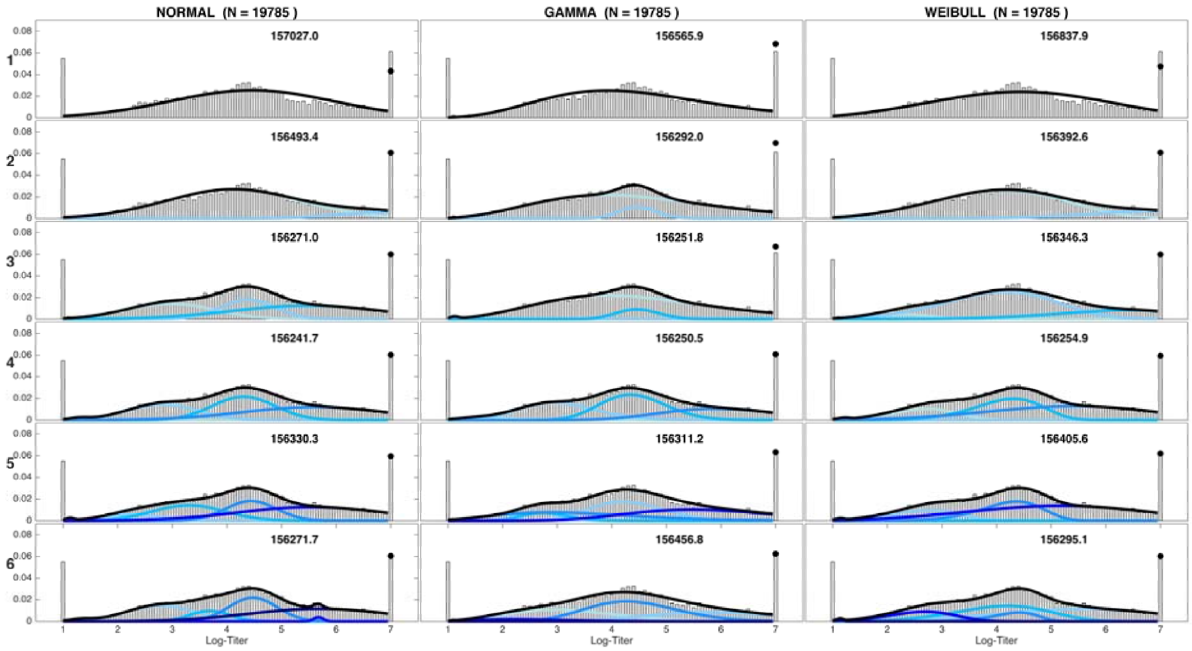

<u>Figure S7:</u> H3N2 titers, all sites all ages. Mixture fits were performed with three different distributional forms, shown in the three columns. The first row of figures shows fits with a single mixture component, the second row of figures shows fits with two mixture components, etc. The number in the top-right corner of each panel is the BIC value for that model fit.

7. Confidence intervals for mixture component estimates

Here, we present confidence intervals for all the mixture components and weights displayed in Figure 3 of the main text (H1N1).

Table S4: Estimates with 95% confidence intervals in parentheses, for mean, variance, and weight parameters, for 1-component to 4-component mixture models of H1N1 titers.

Number of normal components		ALL SITES (N = 19335)	HUE (N = 4390)	KHANH HOA (N = 5053)	HCMC (N = 5753)	DAK-LAK (N = 4139)
1	w_0	0.228	0.254	0.225	0.224	0.209
	w_1	0.772	0.746	0.775	0.776	0.791
	μ_1	3.950 (3.925 – 3.975)	4.014 (3.962 – 4.065)	3.910 (3.865 – 3.956)	4.018 (3.974 – 4.062)	3.840 (3.790 – 3.890)
	σ_1	1.551 (1.533 - 1.570)	1.576 (1.520 - 1.615)	1.513 (1.480 - 1.547)	1.589 (1.558 - 1.622)	1.515 (1.478 - 1.553)
2	w_0	0.228	0.254	0.225	0.224	0.209
	w_1	0.218 (0.205 - 0.230)	0.242 (0.218 - 0.272)	0.230 (0.208 - 0.256)	0.184 (0.169 - 0.200)	0.251 (0.229 - 0.275)
	μ_1	2.462 (2.445 - 2.500)	2.646 (2.550 - 2.750)	2.502 (2.420 - 2.590)	2.236 (2.285 - 2.440)	2.403 (2.330 - 2.470)
	σ_1	0.698 (0.686 - 0.715)	0.763 (0.723 - 0.816)	0.717 (0.684 - 0.760)	0.659 (0.632 - 0.693)	0.673 (0.645 - 0.705)
	w_2	0.555 (0.542 - 0.568)	0.504 (0.475 - 0.530)	0.545 (0.520 - 0.567)	0.592 (0.577 - 0.608)	0.540 (0.517 - 0.565)
	μ_2	4.532 (4.480 - 4.580	4.671 (4.605 - 4.745)	4.504 (4.450 - 4.570)	4.530 (4.492 - 4.567)	4.506 (4.440 - 4.570)
	σ_2	1.393 (1.377 - 1.410)	1.438 (1.435 - 1.442)	1.356 (1.347 - 1.365)	1.428 (1.425 - 1.433)	1.317 (1.297 - 1.335)
3	w_0	0.228	0.254	0.225	0.224	0.209
	w_1	0.396 (0.365 - 0.420)	0.332 (0.282 - 0.400)	0.066 (0.053 - 0.078)	0.348 (0.320 - 0.382)	0.449 (0.340 - 0.482)
	μ_1	2.771 (2.730 - 2.815)	2.857 (2.720 - 3.020)	1.594 (1.500 - 1.690)	2.668 (2.590 - 2.760)	2.766 (2.700 - 2.835)
	σ_1	0.803 (0.815 - 0.843)	0.870 (0.805 - 0.920)	0.297 (0.255 - 0.345)	0.796 (0.761 - 0.837)	0.830 (0.800 - 0.866)
	w_2	0.159 (0.140 - 0.200)	0.042 (0.035 - 0.062)	0.157 (0.148 - 0.167)	0.184 (0.178 - 0.201)	0.254 (0.227 - 0.272)
	μ_2	4.684 (4.658 - 4.710)	4.922 (4.780 - 5.190)	2.753 (2.680 - 2.810)	4.613 (4.586 - 4.643)	4.794 (4.740 - 4.845)
	σ_2	0.434 (0.413 - 0.472)	0.100 (0.075 - 0.170)	0.402 (0.375 - 0.445)	0.467 (0.465 - 0.468)	0.465 (0.432 - 0.505)
	w_3	0.217 (0.155 - 0.270)	0.372 (0.295 - 0.430)	0.552 (0.528 - 0.572)	0.467 (0.456 - 0.477)	0.088 (0.077 - 0.112)
	μ_3	5.571 (5.250- 6.100)	4.948 (4.820 - 5.280)	4.514 (4.420 - 4.610)	5.510 (5.290 - 5.870)	6.520 (6.250 - 6.680)
	σ_3	1.298 (1.065 - 1.430)	1.482 (1.170 - 1.795)	1.325 (1.292 - 1.357)	1.415 (1.230 - 1.500)	0.683 (0.587 - 0.862)
4	w_0	0.228	0.254	0.225	0.224	0.209
	w_1	0.062 (0.056 - 0.069)	0.057 (0.046 - 0.072)	0.060 (0.046 - 0.079)	0.054 (0.044 - 0.068)	0.089 (0.063 - 0.122)
	μ_1	1.576 (1.540 - 1.610)	1.601 (1.530 - 1.680)	1.525 (1.450 - 1.610)	1.522 (1.460 - 1.600)	1.692 (1.580 - 1.810)
	σ_1	0.296 (0.280 - 0.313)	0.274 (0.237 - 0.316)	0.270 (0.233 - 0.318)	0.266 (0.238 - 0.308)	0.370 (0.328 - 0.418)
	w_2	0.322 (0.317 - 0.328)	0.347 (0.317 - 0.370)	0.306 (0.285 - 0.328)	0.290 (0.279 - 0.303)	0.338 (0.300 - 0.358)
	μ_2	2.907 (2.875 - 2.940)	3.052 (2.960 - 3.140)	2.850 (2.780 - 2.920)	2.809 (2.740 - 2.880)	2.952 (3.040 - 2.870)
	σ_2	0.619 (0.609 - 0.630)	0.668 (0.642 - 0.708)	0.565 (0.540 - 0.610)	0.615 (0.602 - 0.639)	0.616 (0.550 - 0.650)
	w_3	0.235 (0.226 - 0.239)	0.166 (0.122 - 0.192)	0.202 (0.179 - 0.225)	0.231 (0.218 - 0.246)	0.278 (0.266 - 0.292)
	μ_3	4.628 (4.590 - 4.660)	4.752 (4.660 - 4.840)	4.483 (4.380 - 4.570)	4.568 (4.500 - 4.630)	4.760 (4.700 - 4.820)
	σ_3	0.512 (0.503 - 0.525)	0.435 (0.360 - 0.500)	0.512 (0.485 - 0.565)	0.516 (0.492 - 0.553)	0.491 (0.474 - 0.517)
	w_4	0.153 (0.128 - 0.191)	0.176 (0.128 - 0.260)	0.207 (0.115 - 0.290)	0.201 (0.141 - 0.260)	0.086 (0.075 - 0.106)
	μ_4	6.068 (5.700 - 6.300)	5.993 (5.410 - 6.320)	5.610 (5.000 - 6.290)	5.806 (5.395 - 6.320)	6.546 (5.395 - 6.700)
	σ_4	1.058 (0.925 - 1.220)	1.162 (0.940 - 1.450)	1.134 (0.820 - 1.350)	1.287 (1.020 - 1.450)	0.665 (0.565 - 0.850)

And below, we show the same confidence intervals for H3N2.

Table S5: Estimates with 95% confidence intervals in parentheses, for mean, variance, and weight parameters, for 1-component to 4-component mixture models of H3N2 titers.

Number of normal components		ALL SITES (N = 19785)	HUE (N = 4519)	KHANH HOA (N = 5384)	HCMC (N = 5784)	DAK-LAK (N = 4134)
1	w_0	0.055	0.066	0.054	0.065	0.028
	w_1	0.945	0.934	0.946	0.935	0.972
	μ_1	4.483 (4.472 – 4.495)	4.459 (4.415 – 4.500)	4.434 (4.398 – 4.468)	4.693 (4.655 – 4.600)	4.292 (4.250 – 4.335)
	σ_1	1.491 (1.483 - 1.499)	1.476 (1.445 - 1.507)	1.467 (1.441 - 1.493)	1.587 (1.560 - 1.615)	1.378 (1.347 - 1.410)
2	w_0	0.055	0.066	0.054	0.065	0.028
	w_1	0.827 (0.817 - 0.837)	0.814 (0.615 - 0.840)	0.485 (0.340 - 0.785)	0.813 (0.762 - 0.833)	0.888 (0.778 - 0.910)
	μ_1	4.140 (4.115 - 4.167)	4.110 (3.880 - 4.190)	3.604 (3.400 - 4.100)	4.315 (4.200 - 4.385)	4.050 (3.910 - 4.120)
	σ_1	1.219 (1.207 - 1.230)	1.198 (1.120 - 1.235)	1.056 (0.980 - 1.190)	1.301 (1.250 - 1.337)	1.150 (1.095 - 1.180)
	w_2	0.118 (0.110 - 0.128)	0.120 (0.095 - 0.320)	0.461 (0.180 - 0.595)	0.122 (0.104 - 0.170)	0.083 (0.065 - 0.195)
	μ_2	6.890 (6.820 - 6.940)	6.841 (5.600 - 7.020)	5.310 (4.950 - 6.240)	7.137 (6.800 - 7.220)	7.016 (5.800 - 7.420)
	σ_2	0.845 (0.810 - 0.905)	0.884 (0.795 - 1.470)	1.334 (1.030 - 1.420)	0.729 (0.640 - 1.040)	1.009 (0.082 - 1.550)
3	w_0	0.055	0.066	0.054	0.065	0.028
	w_1	0.287 (0.270 - 0.303)	0.279 (0.235 - 0.333)	0.405 (0.330 - 0.475)	0.332 (0.278 - 0.410)	0.186 (0.152 - 0.230)
	μ_1	3.003 (2.940 - 3.060)	2.983 (2.800 - 3.185)	3.226 (3.070 - 3.390)	3.267 (3.070 - 3.480)	2.581 (2.420 - 2.770)
	σ_1	0.795 (0.770 - 0.820)	0.775 (0.690 - 0.870)	0.873 (0.810 - 0.945)	0.909 (0.833 - 1.000)	0.613 (0.555 - 0.700)
	w_2	0.225 (0.208 - 0.243)	0.211 (0.160 - 0.263)	0.136 (0.117 - 0.148)	0.153 (0.117 - 0.148)	0.412 (0.357 - 0.480)
	μ_2	4.415 (4.390 - 4.437)	4.400 (4.335 - 4.437)	4.540 (4.490 - 4.585)	4.657 (4.570 - 4.705)	4.168 (4.120 - 4.225)
	σ_2	0.497 (0.478 - 0.520)	0.386 (0.318 - 0.465)	0.386 (0.355 - 0.413)	0.447 (0.390 - 0.530)	0.592 (0.539 - 0.672)
	w_3	0.434 (0.410 - 0.458)	0.443 (0.525 - 0.458)	0.404 (0.350 - 0.480)	0.450 (0.412 - 0.488)	0.374 (0.340 - 0.482)
	μ_3	5.508 (5.420 - 5.596)	5.428 (5.100 - 5.466)	5.607 (5.350 - 5.780)	5.768 (5.430 - 5.913)	5.301 (4.990 - 5.400)
	σ_3	1.371 (1.332 - 1.410)	1.381 (1.482 - 1.410)	1.166 (1.085 - 1.280)	1.409 (1.340 - 1.520)	1.426 (1.410 - 1.500)
4	w_0	0.055	0.066	0.054	0.065	0.028
	w_1	0.008 (0.007 - 0.010)	0.231 (0.378 - 0.295)	0.004 (0.004 - 0.009)	0.018 (0.010 - 0.030)	0.020 (0.001 - 0.042)
	μ_1	1.311 (1.263 - 1.370)	2.803 (2.640 - 3.060)	1.100 (1.082 - 1.300)	1.524 (1.370 - 1.690)	1.505 (1.050 - 1.850)
	σ_1	0.181 (0.215 - 1.158)	0.693 (0.610 - 0.820)	0.001 (0.001 - 0.170)	0.283 (0.207 - 0.370)	0.277 (0.110 - 0.430)
	w_2	0.233 (0.215 - 0.252)	0.042 (0.012 - 0.053)	0.313 (0.305 - 0.405)	0.256 (0.010 - 0.030)	0.186 (0.110 - 0.220)
	μ_2	2.844 (2.780 - 2.910)	3.618 (3.540 - 3.740)	3.118 (2.990 - 3.320)	3.081(2.880 - 3.280)	2.504 (2.240 - 2.650)
	σ_2	0.646 (0.614 - 0.700)	0.158 (0.015 - 0.230)	0.793 (0.695 - 0.850)	0.671 (0.560 - 0.790)	0.593 (0.540 - 0.665)
	w_3	0.308 (0.287 - 0.320)	0.225 (0.160 - 0.305)	0.114 (0.107 - 0.265)	0.274 (0.195 - 0.340)	0.453 (0.370 - 0.630)
	μ_3	4.332 (4.298 - 4.367)	4.418 (4.332 - 4.438)	4.515 (4.445 - 4.600)	4.563 (4.448 - 4.660)	4.141 (4.030 - 4.412)
	σ_3	0.572 (0.543 - 0.610)	0.341 (0.320 - 0.373)	0.359 (0.330 - 0.540)	0.563 (0.477 - 0.765)	0.682 (0.580 - 0.860)
	w_4	0.397 (0.378 - 0.402)	0.436 (0.410 - 0.505)	0.514 (0.370 - 0.560)	0.387 (0.280 - 0.480)	0.333 (0.265 - 0.460)
	μ_4	5.634 (5.615 - 5.700)	5.484 (5.250 - 5.535)	5.256 (5.120 - 5.680)	6.006 (5.448 - 6.530)	5.422 (5.120 - 5.780
	σ_4	1.328 (1.303 - 1.321)	1.361 (1.285 - 1.440)	1.324 (0.890 - 1.420)	1.300 (1.020 - 1.510)	1.422 (1.210 - 1.580)

8. Confidence intervals for pandemic attack rate

In the fourth paragraph of the Results section of the main paper, for subtype H1N1, we present evidence showing that the interpretation of w_4 as the fraction of the population that was recently infected, and the interpretation of w_3 as the fraction of the population that was non-recently infected, are consistent with post-pandemic observations in Vietnam in 2010.

Table S6: Estimates and confidence intervals for weight parameters in post-pandemic (January to June 2010) analysis.

Age Group	Number of Samples	w_4 (95% CI)	w_3 (95% CI)
0.5 – 9	161	0.138 (0.117–0.169)	0.160 (0.146–0.174)
10 – 19	355	0.226 (0.200–0.268)	0.216 (0.182–0.234)
20 – 44	871	0.085 (0.070–0.108)	0.230 (0.227–0.393)
≥45	439	0.157 (0.135–0.205)	0.196 (0.169–0.221)

The confidence intervals for these ‘recent attack rates’ and ‘non-recent attack rates’ are presented above, and the w_4 column is consistent with what has been measured for the 2009 pandemic in Vietnam and other parts of Asia.

Note that some of the confidence intervals are quite non-symmetric. All confidence intervals were checked manually to ensure that the appropriate weight parameter was associated with the appropriate mean (category) during the optimization and profiling process.

9. Additional Figures and Tables

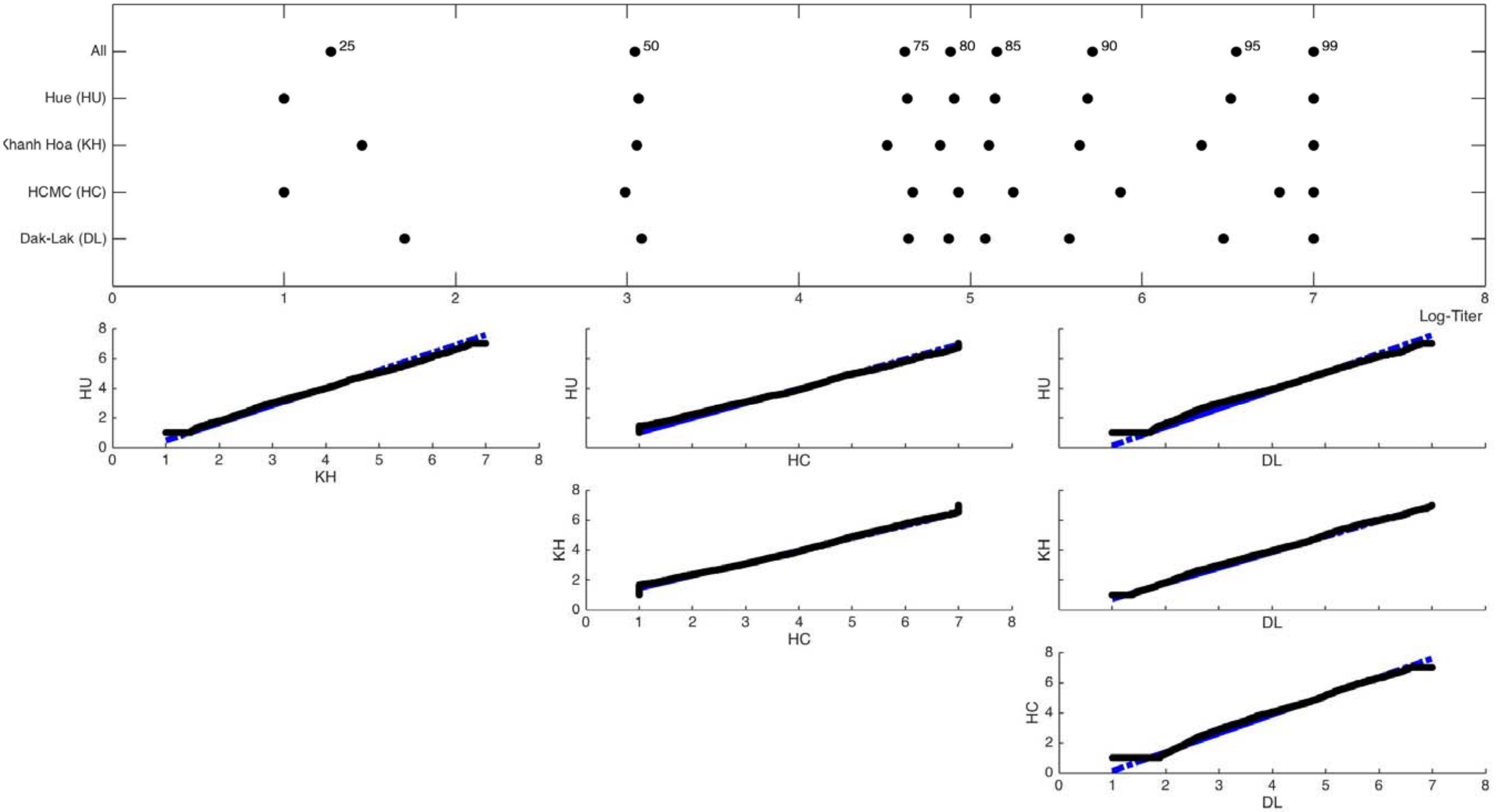

Figure S8: **Top:** Quantile plot showing the 25th, 50th, 75th, 80th, 85th, 90th, 95th, and 99th percentiles of log-titer for 2009 H1N1 (log-titer on *x*-axis) for the aggregated data and the individual collection sites (*y*-axis). **Bottom:** quantile-quantile plots for pairwise comparison among the four collection sites (all age groups included; not re-weighted by province population). When the two datasets contain different numbers of samples, the plotting quantiles were calculated based on the smaller dataset. Blue lines are the extrapolated straight line connecting the first and third quartiles of the smaller dataset.

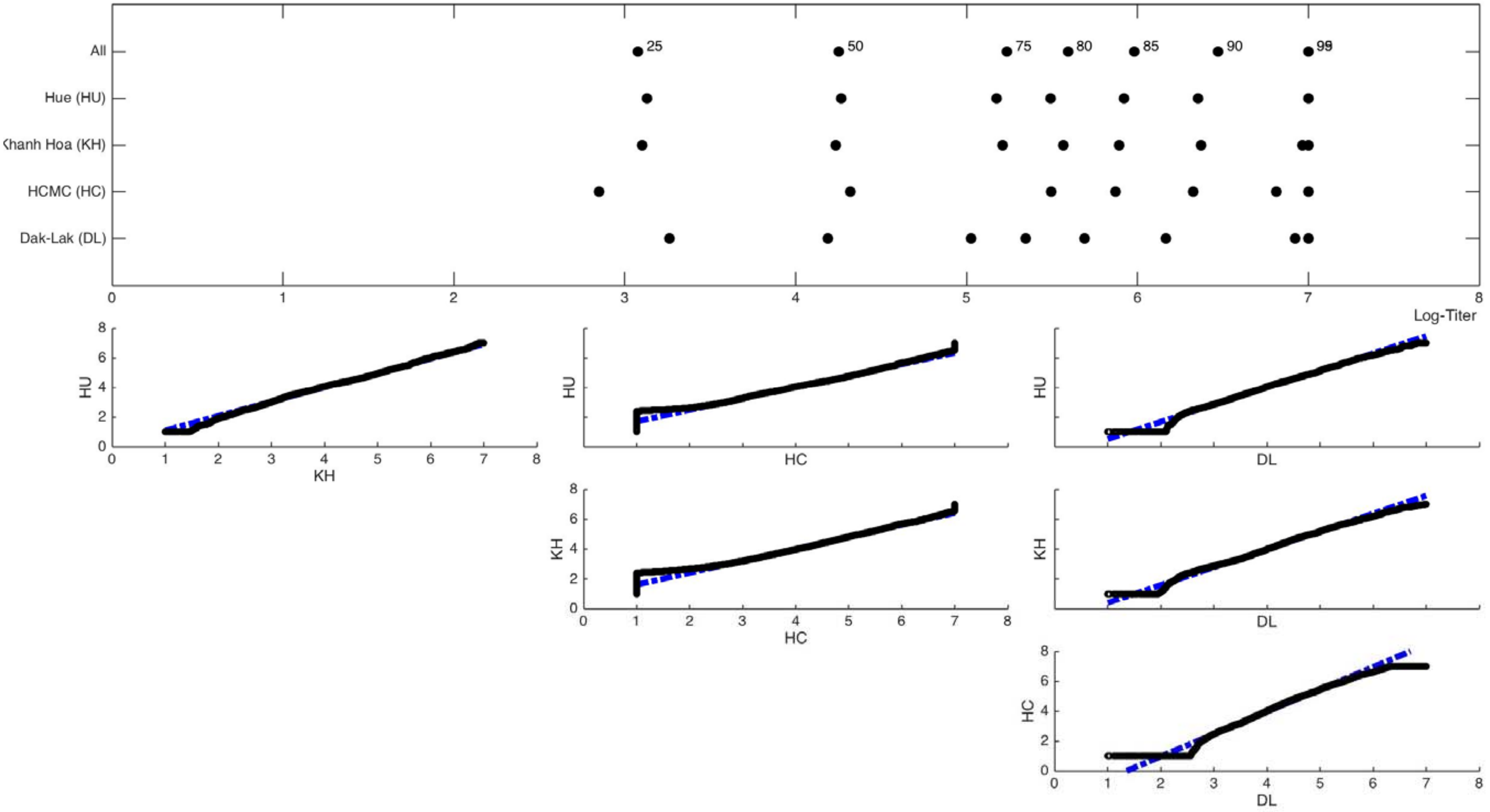

<u>Figure S9:</u> **Top:** Quantile plot showing the 25th, 50th, 75th, 80th, 85th, 90th, 95th, and 99th percentiles of log-titer of H3N2 (geometric mean titer of 2009 and 2011 strains). Log-titer is shown on the *x*-axis and collection sites are shown on the *y*-axis. **Bottom:** quantile-quantile plots for pairwise comparison among the four collection sites (all age groups included; not re-weighted by province population). When the two datasets contain different numbers of samples, the plotting quantiles were calculated based on the smaller dataset. Blue lines are the extrapolated straight line connecting the first and third quartiles of the smaller dataset.

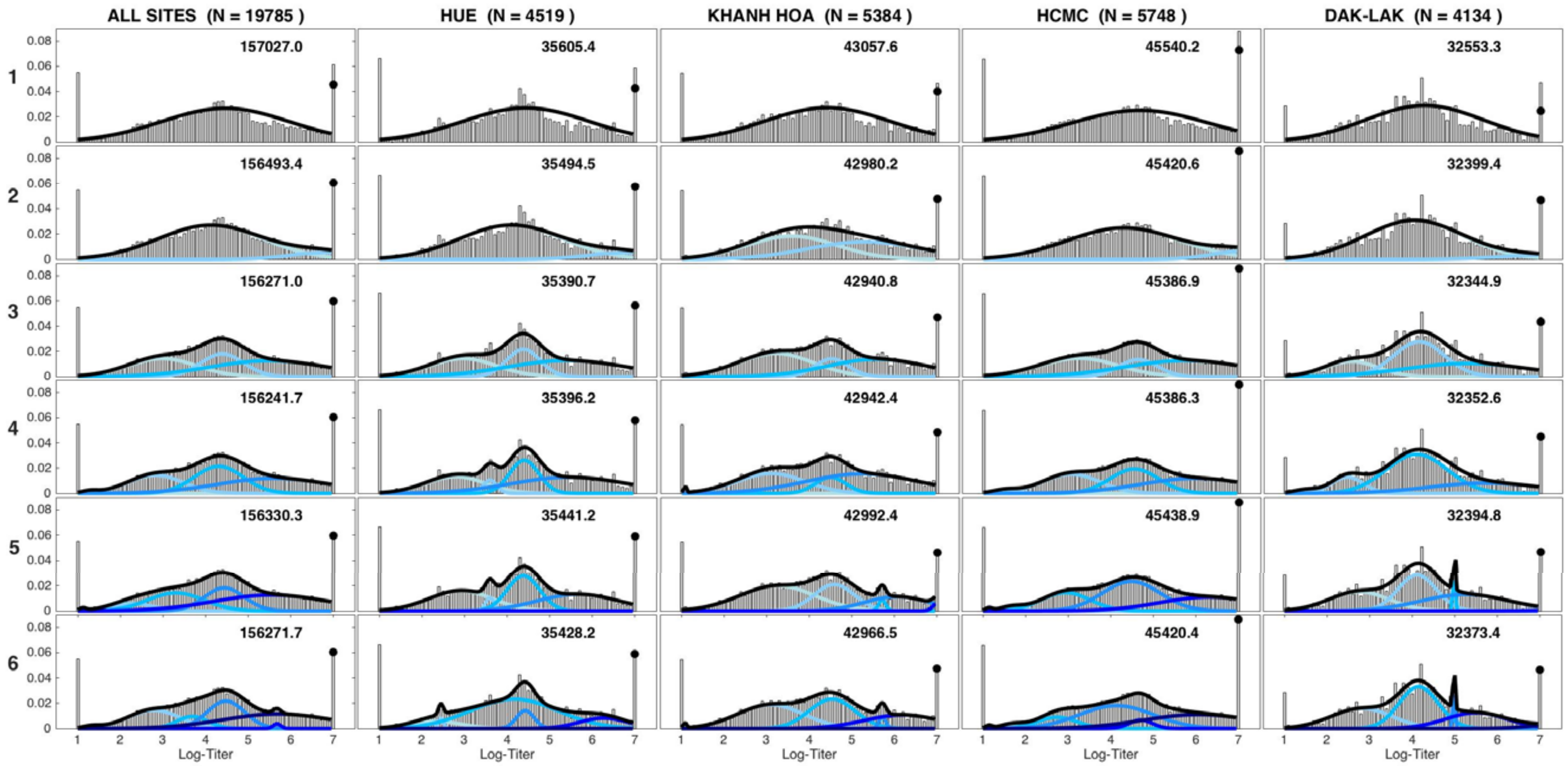

Figure S10: Histograms of antibody titers to H3N2 (GMT of 2009 and 2011 strains), showing fit results for mixture models with different number of normal components (top to bottom, the label to the left of the *y*-axis is the number of mixture components) and grouped by collection sites (left to right). Histograms are weighted to adjust for age and gender according to the Vietnam national housing census in 2009 for each of the four collection sites. The blue lines in each panel are the normalized probability density functions of the component distributions whose intensity was ranked in order of increasing μ. The black lines show the full mixture distribution density, and the black dots are the estimated cumulative distribution of the mixture models at 7.0 (titer of 1280). The numbers in the upper right corner of each panel are the BIC scores of the model fits.

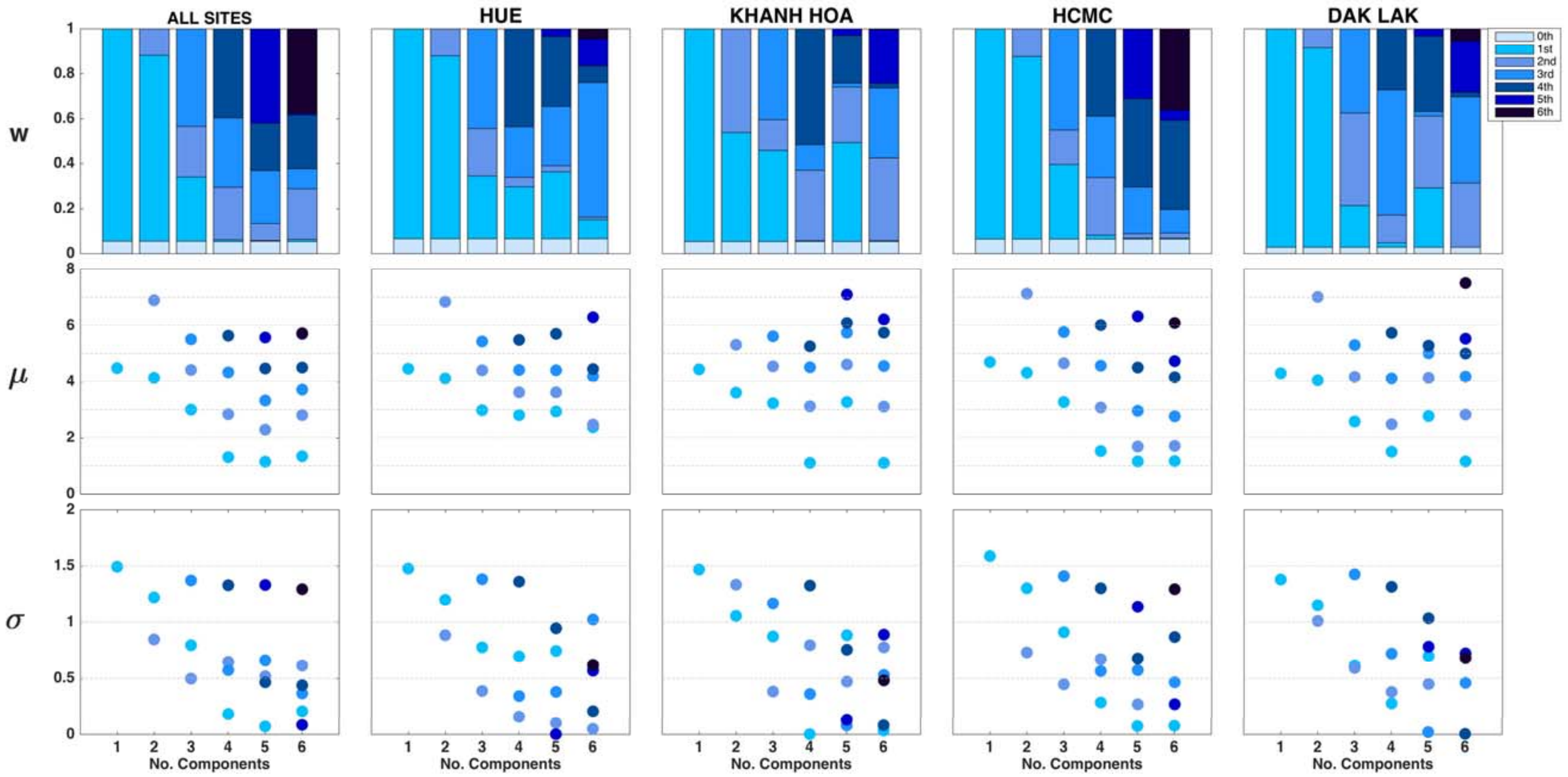

Figure S11: Visualization of model selection process for H3N2 titer distributions from Figure S8. The *y*-axes show the fitted values of w_i (mixture weights), μ_i(means), and σ_i (standard deviations). Components' shades were ranked from lightest to darkest in increasing order of μ. In the top panel, the "0th component" represents the point mass w_0 placed at 20 for titers below the lower detection limit of 20. Note that for five or six components, in many cases, the weights or standard deviation parameters are close to zero; for some cases, two of the inferred mean parameters are very close to each other.

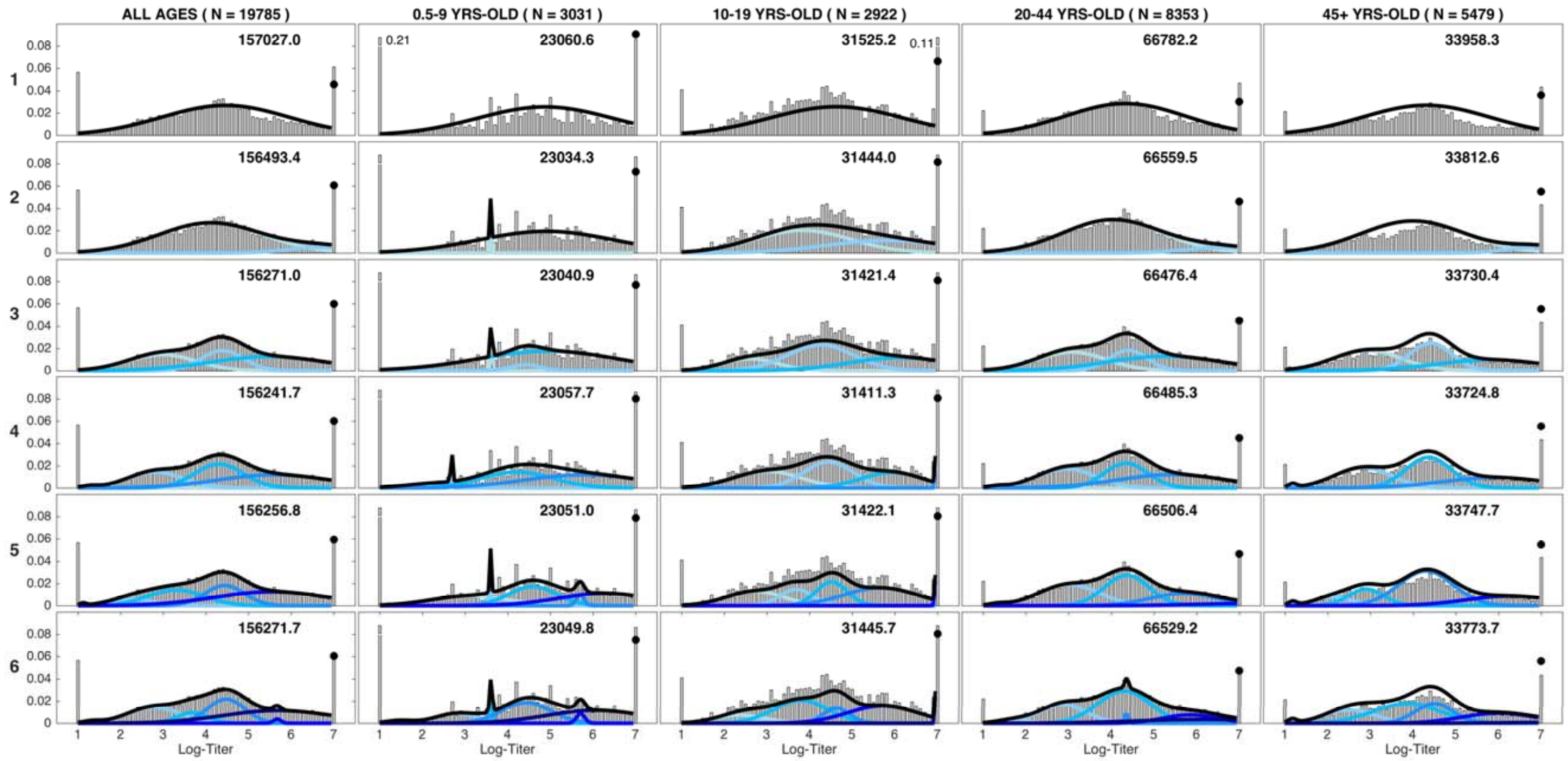

<u>Figure S12:</u> Titer histograms for H3N2 and fit results for mixture models with different number of components (label on the left is the number of mixture components) and grouped by different age groups. Histograms are weighted to adjust for age and gender according to the Vietnam national housing census in 2009. The numbers in the upper right corner of each panel are the fitted BIC scores of the respective model. For each panel, the blue lines are the normalized probability density of the component distributions whose intensity from lightest to darkest was ranked in order of increasing μ. Black lines are the total mixture distribution density; and the black dots are estimated probability weight of the mixture model for titers ≥ 7.0.

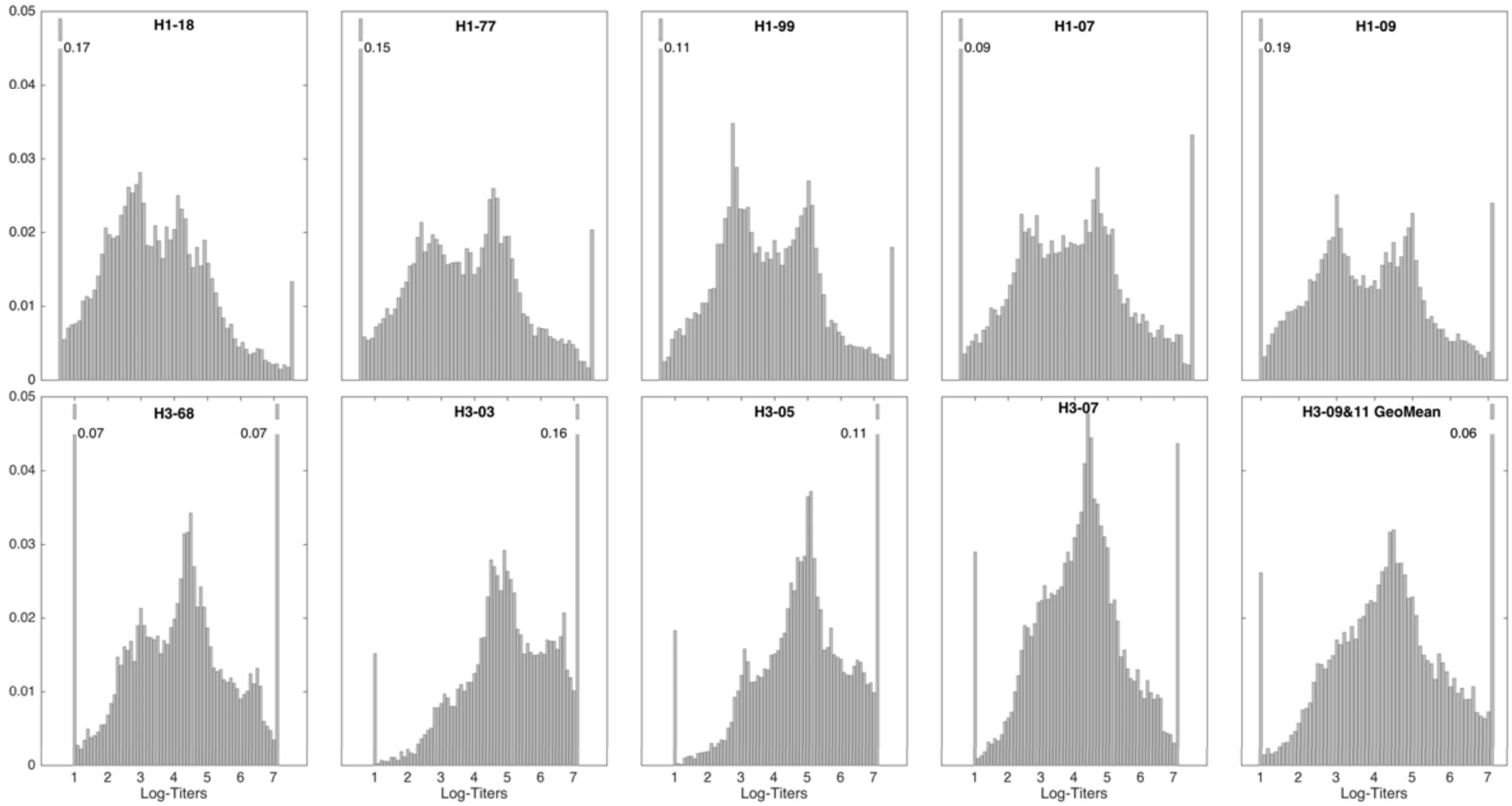

<u>Figure S13:</u> Antibody titer histograms for n = 20,152 individuals, plotted for H1N1 strains (top panels) and H3N2 strains (bottom panels). The fractions of individuals with titers below the detection limit of 20 and above 1280 that were out of the plotting ranges are given next to the respective bar. Histograms were weighted to adjust for age and gender according to the Vietnam national housing census in 2009 for the four collection sites.

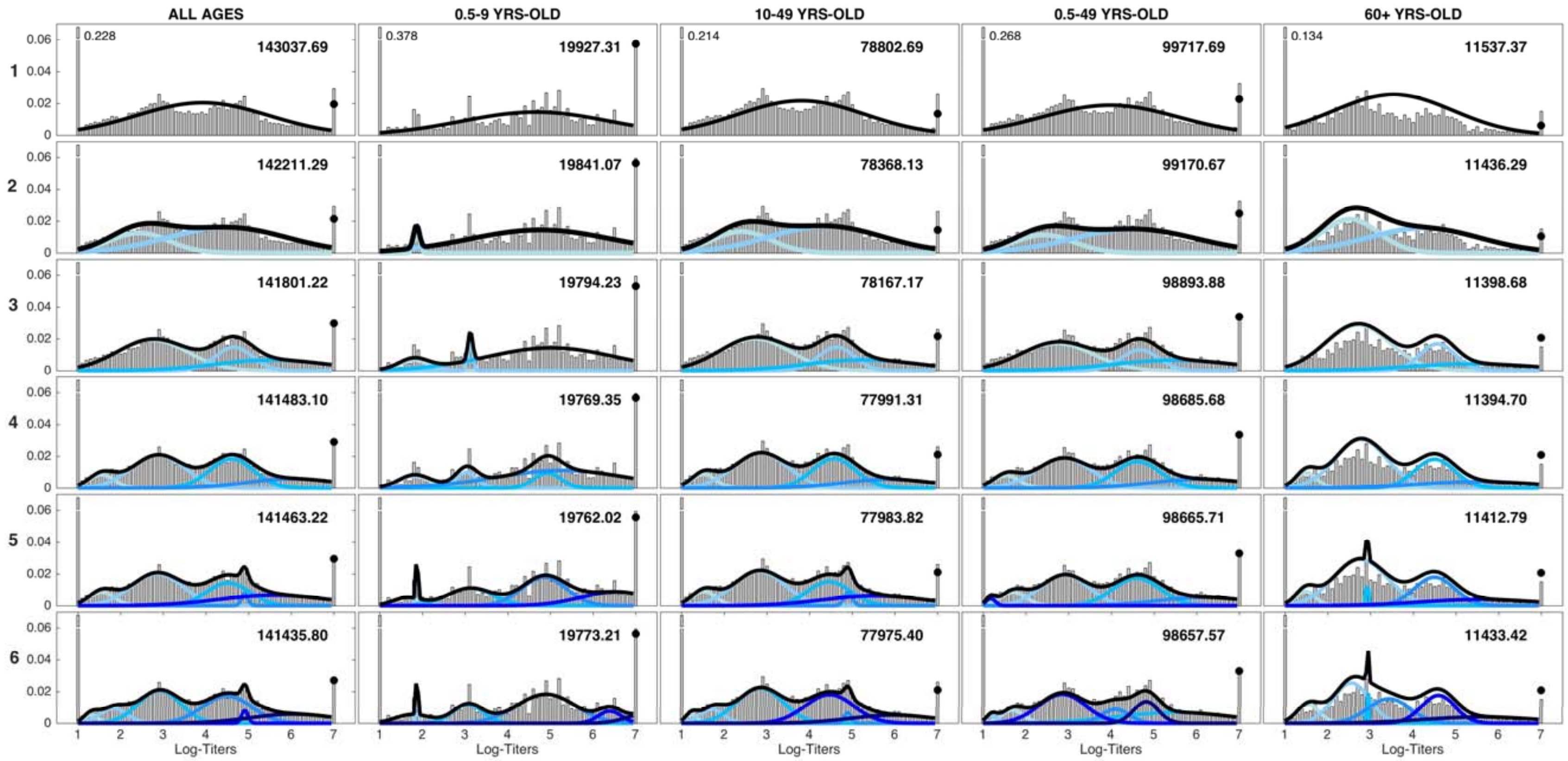

Figure S14: Similar to Figure 5 in the main text, but age groupings are different. Individuals in the 0.5-49 age group were born in 1960 or later and would have been exposed to two lineages of H1N1: the 1977 lineage and the 2009 pandemic lineage. Individuals in the 60+ age group were born in 1953 or earlier and are likely to have been exposed to three H1N1 lineages: the 1918-1957 lineage, the 1977 lineage, and the 2009 pandemic lineage. The BIC comparisons (see also Table S6) shows that 3 components is the best fit for 60+ and that 4 components is the best fit for 0.5-49. However, the difference between the 3-component fits and 4-component fits here – as in all other groupings that have been looked at – is that the 4-component fit includes an extra small peak in the seronegative titer range, covering titers between 20 and 30. There does not appear to be a reason why the different lineage-exposure history would produce this extra peak for seronegative individuals.

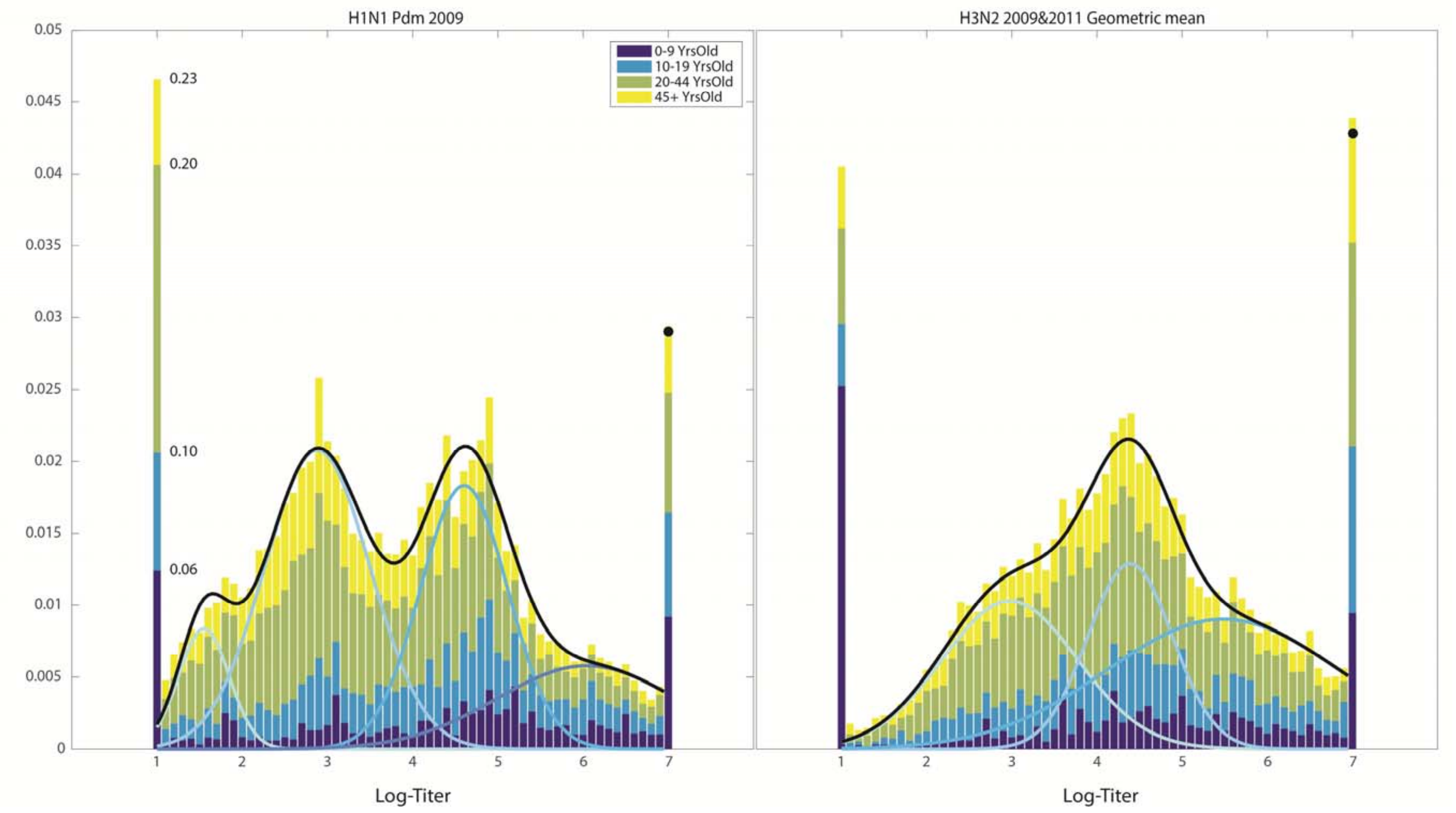

Figure S15: An additional visualization of age structure in the titer distribution. Here, the four-component model is shown for H1N1 and the three-component model is shown for H3N2. The individual histogram bins (0.1 width on a $\log_2$-scale) are displayed as a stacked bar graph with different age groups corresponding to different colors. Note that for H1N1, the left-hand bar at a log-titer of one is shown on a separate scale. There are no clear age patterns in these titer distributions suggesting that certain mixture components correspond to certain age groups.

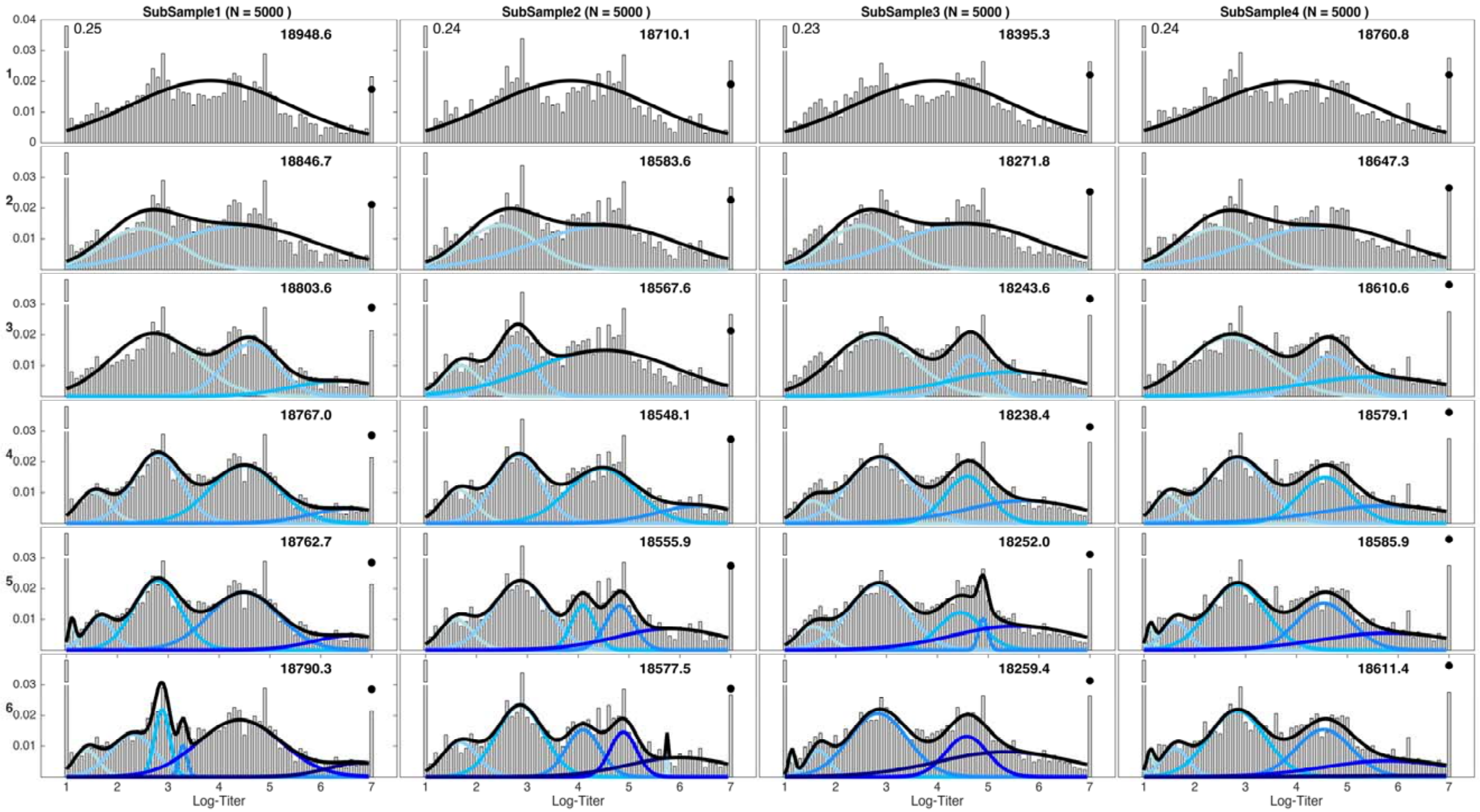

Figure S16: As Figure 2 in the main text, but here four random sub-samples were drawn (with replacement) according to the age-group proportions and age bands in the general population, as shown in Figure S5. Four or five components is the best explanatory model, according to BIC. However, the component means, variances, and weights are not the same across sub-samples when five components are used.

Table S7: Change in BIC scores as the number of normal distributions in the mixture increase from one to six for H3N2, for the aggregated data as well as the individual collection sites. The values of the negative log-likelihood were weighted to adjust for age and gender according to the Vietnam national housing census in 2009. The first row shows the exact values of the negative log-likelihood. Bold numbers represent the mixtures with the best BIC. **NB:** Even though the BIC is designed to take sample size into account in selecting the best fitted models, the large number of data points in the aggregate data (~20,000) still caused the BIC to select six components as the best model for H1N1 (Figure 2) and four components as the best model for H3N2 (Figure S8). Thus, BIC is imperfect and cannot be used as the sole criterion for model selection.

		ALL SITES (N = 19785)	HUE (N = 4519)	KHANH HOA (N = 5384)	HCMC (N = 5784)	DAK LAK (N = 4134)
	SumNegLLH (C =1)	78498.68	17790.08	21515.90	22757.09	16264.15
Δ in num components	1 to 2	-533.59	-110.90	-77.42	-119.56	-153.85
	2 to 3	-222.45	-103.79	-39.32	-33.62	-54.56
	3 to 4	-29.26	5.47	1.52	-0.60	7.74
	4 to 5	15.05	15.82	18.17	12.95	-2.89
	5 to 6	14.88	16.15	5.95	21.13	23.64

Table S8: Change in BIC scores as the number of normal distributions in the mixture increase from one to six for H3N2, for different age groups as recommended by CONCISE. The values of the negative log-likelihood were weighted to adjust for age and gender according to the Vietnam national housing census in 2009. The first row shows the exact values of the negative log-likelihood. Bold numbers represent the mixtures with the best BIC.

		ALL AGES (N = 19785)	0-9 Yrs-Old (N = 3031)	10-19 Yrs-Old (N = 2922)	20-44 Yrs-Old (N = 8353)	45+ Yrs-Old (N = 5479)
SumNegLLH (C=1)		78498.68	11518.25	15750.62	33377.53	16966.21
Δ in num components	1 to 2	-533.59	-26.27	-81.21	-222.69	-145.63
	2 to 3	-222.45	6.62	-22.60	-83.06	-82.27
	3 to 4	-29.26	16.77	-10.09	8.94	-5.55
	4 to 5	15.05	-6.69	10.78	21.10	22.92
	5 to 6	14.88	-1.14	23.64	22.79	25.97

Table S9: Change in BIC scores as the number of normal distributions in the mixture increase from one to six for H1N1, for different age groups as recommended by CONCISE. The values of the negative log-likelihood were weighted to adjust for age and gender according to the Vietnam national housing census in 2009. The first row shows the exact values of the negative log-likelihood. Bold numbers represent the mixtures with the best BIC.

		ALL AGES (N = 19335)	0-9 Yrs-Old (N = 2988)	10-19 Yrs-Old (N = 2876)	20-44 Yrs-Old (N = 8116)	45+ Yrs-Old (N = 5355)	0-49 Yrs-Old (N = 15168)	60+ Yrs-Old (N = 2152)
SumNegLLH (C=1)		71504.04	9951.65	14564.33	30125.89	16100.72	49844.40	5757.17
Δ in num components	1 to 2	-1670.41	-86.24	-77.86	-345.89	-238.01	-547.01	-101.08
	2 to 3	-837.75	-46.85	-58.34	-206.63	-90.59	-276.79	-37.61
	3 to 4	-653.85	-24.88	-50.71	-134.61	-58.24	-208.20	-3.98
	4 to 5	-57.35	-7.33	4.76	-7.33	13.75	-19.98	18.09
	5 to 6	-72.45	11.20	-0.88	30.17	17.70	-8.14	20.63
